# Continuous monitoring of blood–interstitial fluid intercompartmental molecular kinetics in freely moving animals

**DOI:** 10.1126/sciadv.aed1895

**Published:** 2026-05-20

**Authors:** Yihang Chen, Qitao Hu, Jean Won Kwak, David Zakharian, Jenny Ji, Du Liu, Deepak Gopalan, Caoimhe Lyons, Max Yates, Steven Yee, Han Cui, Michael Eisenstein, Hyongsok Tom Soh

**Affiliations:** ^1^ Department of Materials Science and Engineering, Stanford University, Stanford, CA 94305, USA.; ^2^ Department of Radiology, Stanford University, Stanford, CA 94305, USA.; ^3^ Department of Electrical Engineering, Stanford University, Stanford, CA 94305, USA.; ^4^ Department of Bioengineering, Stanford University, Stanford, CA 94305, USA.; ^5^ Wu Tsai Neurosciences Institute, Stanford University, Stanford, CA, USA.

## Abstract

Accurate health monitoring depends on continuous assessment of biochemical markers across multiple body compartments. While blood remains the clinical gold standard, interstitial fluid (ISF) presents a minimally invasive alternative that reflects local tissue physiology, although its biomarker kinetics often differ from blood. It is currently impossible to achieve simultaneous, continuous monitoring of both compartments, limiting insight into their dynamic relationship. To address this, we present BICEP (blood-ISF continuous-sensing electrochemical probe), a wireless electrochemical platform for continuous dual-compartment monitoring of analytes in blood and ISF. The BICEP system incorporates microfabricated soft sensors with biofouling-resistant aptamer interfaces, paired with a wearable potentiostat for remote configuration and readout. Using this system, we continuously tracked kanamycin signal in both anesthetized and freely moving rats, revealing distinct kinetic profiles and individualized temporal delays between blood and ISF signals. These findings confirm that ISF is not merely a proxy for blood but exhibits unique molecular kinetics influenced by individual variability and physiological state. This platform advances personalized health monitoring by providing integrated, dynamic correlation of blood and ISF biomarkers, deepening understanding of their physiological interplay under real-life conditions and paving the way for broader use of wearable ISF-based biosensors.

## Teaser

Soft wireless sensors track drug signals in blood and tissue fluid, revealing how molecules move between body compartments.

## INTRODUCTION

Blood-based analysis is a fundamental component of modern medical diagnostics and health assessment, but blood sampling is an invasive process that can be stressful and unpleasant for patients (*1*). Interstitial fluid (ISF) offers a promising alternative in this context, as it is more accessible than blood while also providing more physiologically relevant molecular information than other biofluids such as sweat, saliva, or tears (*2*–*7*). ISF is generally recognized as the extravascular fluid derived from blood plasma via capillary filtration (*8*), and comparative studies have shown that ISF and blood exhibit similar molecular biomarker profiles, where most small molecules (<3 kDa) and over 90% of proteins found in blood are also detectable in ISF (*2*, *9*–*12*). However, the physiological value of ISF signals for diagnostic purposes depends on a deeper understanding of the kinetics of molecular transport between the blood and ISF compartments for clinically relevant analytes.

Currently, the interplay between blood and ISF under real-world conditions remains poorly understood. This relationship appears to be dynamic and context dependent, varying across analytes, individuals, and physiological states such anesthesia and consciousness (*13*–*15*). ISF-based continuous glucose monitoring can be paired with blood-based glucometers to reveal correlations between glucose levels in these two compartments, albeit with substantial delays in the ISF signal—ranging from 0 to 45 min among subjects (*16*–*18*). However, glucose is currently the only analyte for which such profiling can be performed in a continuous fashion and most intercompartmental comparisons for other analytes have been performed by assessing samples collected at various time points using molecular profiling techniques such as transcriptomic analysis via RNA sequencing or proteomic/metabolomic analysis via liquid chromatography–mass spectrometry (*9*, *19*, *20*). These approaches are invasive, labor intensive, and limited by poor temporal resolution. These episodic and infrequent measurements also fail to capture critical pharmacokinetic transitions and overlook dynamic shifts in the correlations between compartments in response to changing physiological or behavioral states (*21*). At present, there is no technology that is capable of delivering synchronized, high-resolution molecular measurements in situ from both blood and ISF in live, freely moving subjects. In the absence of such capabilities, it has remained difficult to characterize molecular transport between these two compartments, presenting a major impediment to efforts to transition from blood testing to ISF-based measurements in clinical settings.

In this work, we present a fully integrated system, BICEP (blood-ISF continuous-sensing electrochemical probe), that enables wireless, simultaneous, continuous molecular monitoring in both the blood and ISF of freely moving subjects. This platform uses a soft, implantable microfabricated sensor shank coupled to a wearable, plug-and-play wireless potentiostat for real-time square-wave voltammetry (SWV) data acquisition and analysis. To enable stable, continuous biochemical monitoring within biological environments, the sensor shank uses a nanostructured aptamer-based electrochemical interface, as detailed in our previous work (*22*). As proof of concept, we used BICEP to monitor kanamycin pharmacokinetics in the bloodstream and ISF of individual rats, capturing transport delays and revealing insights into intercompartment molecular kinetics under both anesthetized and conscious, freely moving conditions. Our results confirm that the interplay between blood and ISF is not static but rather is dynamically modulated by both cross-sectional variables (e.g., body weight) and longitudinal physiological states (e.g., level of consciousness). These findings experimentally confirm that the ISF is not a simple proxy for blood but is actually a distinct compartment with complex coupling to the vasculature. Hence, BICEP offers a useful platform for dissecting these intercompartment molecular dynamics, laying critical groundwork for advancing the development of ISF-based diagnostics and a new generation of personalized health monitoring systems.

## RESULTS

### BICEP design enables correlation of blood and ISF molecular kinetics

**Fig. 1.**
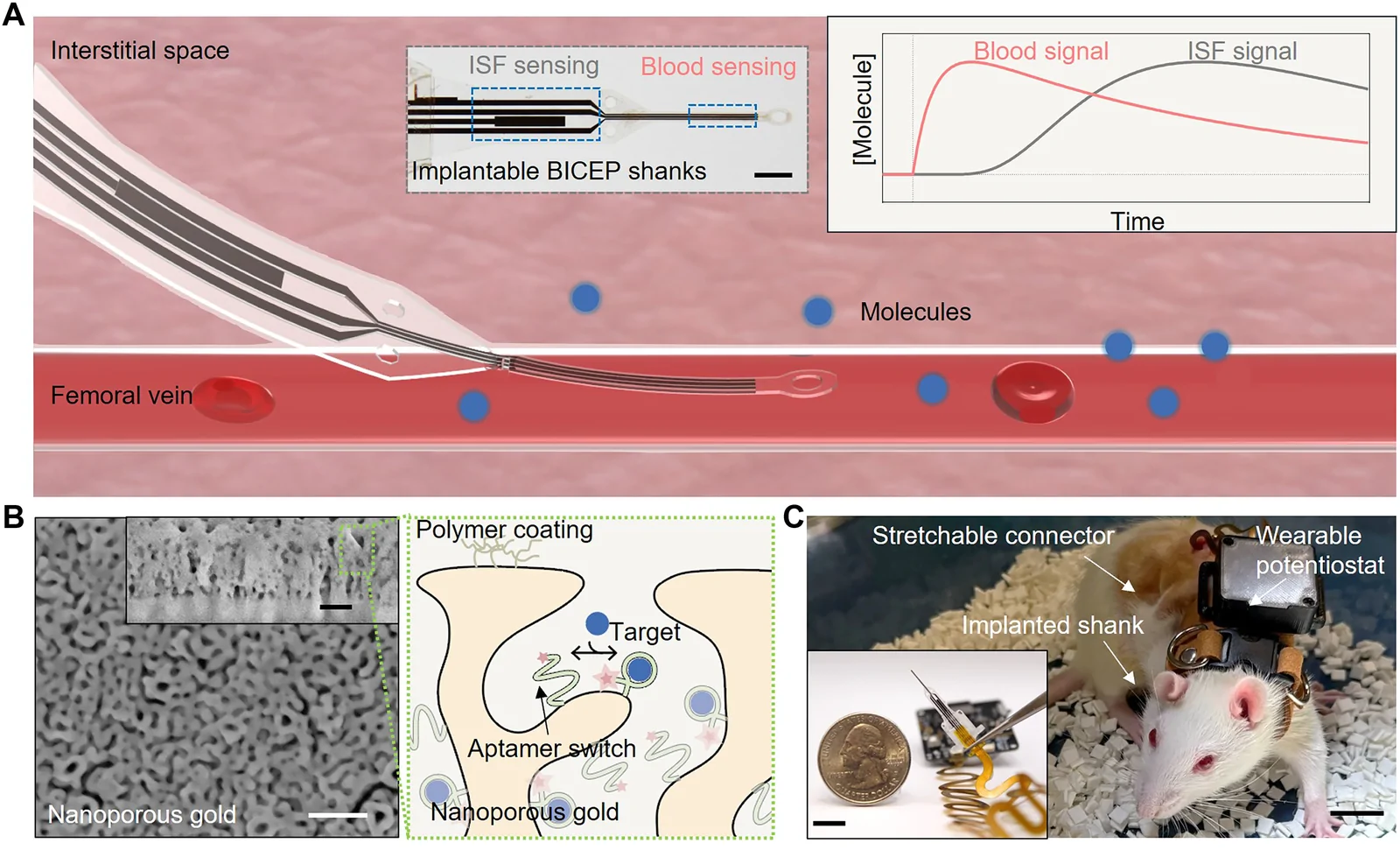
Overview and design of the BICEP. (**A**) The BICEP system enables simultaneous, real-time, wireless molecular monitoring of both the blood and ISF in a freely moving rat. The lefthand inset shows the sensor shank, which features a blood sensor implanted in the rat femoral vein and an ISF sensor interfacing with surrounding interstitial tissue. Scale bar, 2 mm. The righthand inset illustrates simultaneous readouts collected from the two compartments in real time. (**B**) Left: Focused ion beam scanning electron microscopy (SEM) images of nanoporous gold electrodes from top and cross-sectional (inset) views. Scale bar, 100 nm. Right: Magnified schematic of the aptamer-based electrochemical interface, where aptamers immobilized within the 3D nanoporous gold surface reversibly bind target analytes, enabling continuous biochemical monitoring in both blood and ISF. (**C**) Photograph of a freely moving rat undergoing real-time continuous blood-ISF monitoring with the BICEP system. The wireless electrochemical board is mounted on an animal jacket and connected subcutaneously to the implanted sensor shank via a stretchable flexible printed circuit (FPC) cable. Scale bar, 2 cm. Inset: BICEP system next to a US quarter. Scale bar, 1 cm.

BICEP integrates multiple technological advances to enable continuous and simultaneous monitoring of blood-ISF molecular kinetics in vivo (Fig. 1A). Our system uses a soft, microfabricated SU8 sensor shank, which provides stable and spatially confined access to both blood and ISF. The probe shank is designed to match the geometry of the rat femoral vein (0.84 ± 0.30 mm), with distinct regions for blood sensing and ISF sensing (fig. S4). The blood sensing section (6.8 mm by 0.5 mm by 0.04 mm) is smaller than a 26-gauge intravenous catheter and is inserted directly into the femoral vein, while the larger ISF-sensing section is larger (11 mm by 3 mm by 0.04 mm) and trapped against the vessel wall, preventing blood contact and interfacing with adjacent interstitial tissue. Suture holes enable precise fixation within the vasculature and surrounding tissue, maintaining stable contact with both compartments during both anesthesia and freely moving conditions. Continuous biochemical detection is achieved via electrochemically modified aptamer switches that undergo reversible, binding-induced conformational changes, producing a measurable shift in electrical current that enables real-time quantification of target analytes (Fig. 1B) (*23*–*25*). These aptamer switches are integrated into a biomimetic three-dimensional (3D) nanoporous gold architecture, which emulates the structural complexity of epithelial microvilli and physically sequesters the sensing interface from interfering macromolecules. A hyperbranched polymer coating, inspired by mucosal glycans, further enhances antifouling by insulating the surface from degradation and nonspecific adsorption (*22*, *26*). As demonstrated in previous work, this combination of nanoporous architecture and a polymer barrier provides synergistic protection against fouling and signal drift and degradation while allowing efficient analyte transport, preserving signal fidelity within complex biological environments over extended durations (*22*).

Our BICEP system also incorporates a lightweight, compact wearable unit that enables untethered molecular monitoring in freely moving animals (Fig. 1C). This wireless unit integrates plug-and-play input/output (I/O) for biosensor shank connection, a miniaturized potentiostat for electrochemical signal acquisition, and a Bluetooth low energy (BLE) module for wireless data transmission. A custom graphical user interface (GUI) allows remote monitoring and control in real time. With a compact form factor (3 cm by 3 cm by 1 cm) and low mass (15.3 g with battery), the unit is designed to be worn by rats (average mass, 200 to 500 g), allowing minimally disruptive operation in naturalistic settings. Together, the implantable aptamer switch–mounted sensor shank and wearable potentiostat communication unit make BICEP suitable for correlating molecular kinetics between blood and ISF in freely moving animals.

### Continuous dual-compartment monitoring using BICEP shank

**Fig. 2.**
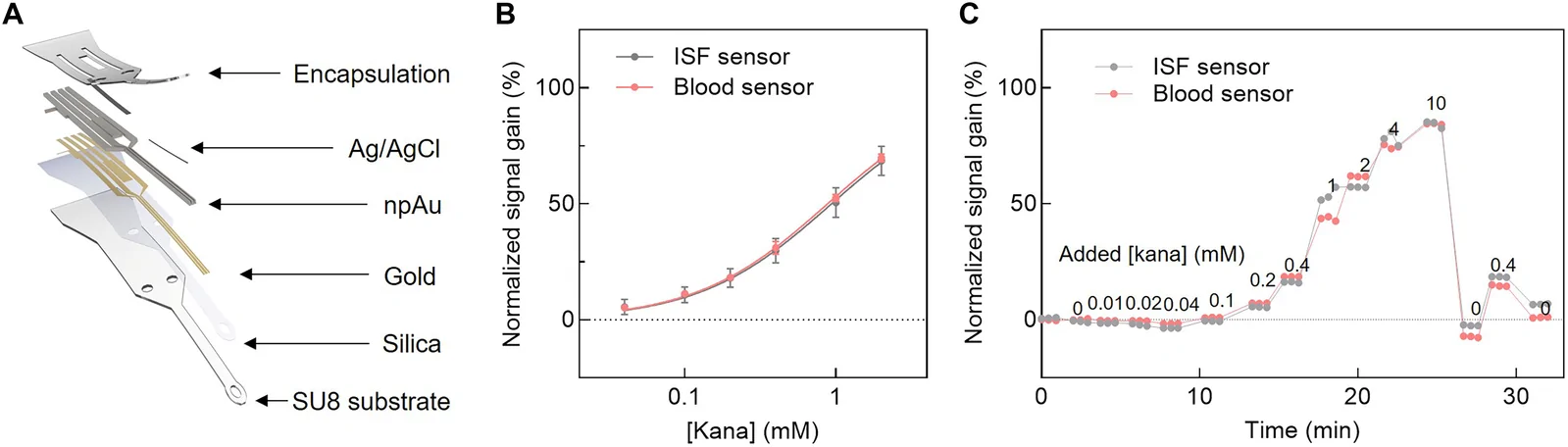
Characterization of the BICEP sensor shank for dual-compartment, continuous molecular monitoring in vitro. (**A**) Exploded-view illustration of the soft, microfabricated BICEP sensor shank with exposed nanoporous gold (npAu) electrode for dual-compartment sensing. (**B**) Calibration curves from a kanamycin (kana)-sensing BICEP shank using SWV readouts from both the blood and ISF sensors. Signals were normalized to the maximum binding signal (*B*_max_) based on Langmuir isotherm fitting. Data points and error bars represent the mean and SD, respectively, for three independent sensor shanks, each averaged over three consecutive measurements at 200-Hz SWV. (**C**) Real-time dual-monitoring of kanamycin spike-in and washout in PBS using the integrated blood and ISF sensors on the same BICEP shank at 50-Hz SWV.

We fabricated the 40-μm-thick soft BICEP shank on a flexible SU8 substrate, incorporating the implantation geometry and biosensing nanostructures described above (Fig. 2A, fig. S5, and fabrication details provided in Materials and Methods). Briefly, gold conducting lines were patterned and overlaid with a 300-nm-thick 3D nanoporous gold layer (pore size, 11.26 ± 1.497 nm) (*22*, *27*) and then passivated with SU8. A SiO_2_ layer deposited on the SU8 substrate to both tune probe stiffness for surgical manipulation and protect against microcrack formation during downstream nanoporous etching. Following liftoff and silver electroplating of the reference electrode, the probe was completed with four I/O connection pins: a shared Ag/AgCl reference electrode, a blood working electrode, an ISF working electrode, and a shared nanoporous gold counter electrode, compatible with commercial flexible flat cable (FFC) or custom stretchable flexible printed circuit (FPC) cables (fig. S6). To functionalize the shank, we converted an existing kanamycin aptamer (26 nt; $K_{D}$ dissociation constant ~ 0.5 mM) into an electrochemically active, conformation-changing aptamer switch as described previously (*22*), with a methylene blue group at its 3′ end and an alkane thiol group at its 5′ end for coupling to the nanoporous gold electrode surface via thiol-gold interactions. The aptamer-loaded nanoporous gold surface was coated with thiolated polyethylene glycol (PEG), followed by passivation with 6-mercapto-1-hexanol (MCH) to backfill gaps between polymer chains and aptamers, minimizing fouling and nonspecific redox reactions, as established in our prior work (*22*).

We next characterized the sensitivities of the blood and ISF sensors integrated on a single BICEP shank. Electrical connections were mechanically shielded within a 3D printed connector and secured via anisotropic conductive film bonding to an FFC, which interfaces with a custom printed circuit board adapter and a commercial multiplexed potentiostat. Binding curves were quantified as SWV signal gain at 200 Hz in response to a range of kanamycin concentrations in phosphate-buffered saline (PBS) and fitted to the Langmuir isotherm model. Both the blood and ISF sensors on the same BICEP shank exhibited sufficient sensitivity to kanamycin (Fig. 2B), with a fitted $K_{D}$ of ~0.9 mM, highlighting the system’s capacity for quantitative analyte detection in both compartments. Moreover, the BICEP design is inherently modular, such that the aptamer component can readily be replaced with alternative switch sequences that respond to other analytes. To demonstrate this, we evaluated BICEP shanks functionalized with aptamers targeting the chemotherapeutic agent doxorubicin (*28*) (28 nt; $K_{D}$ ~ 2.1 μM) and the antibiotic vancomycin (*29*) (45 nt; $K_{D}$ ~ 70 μM) with only MCH coating, following the passivation optimization process discussed in our prior work (*22*). The resulting sensors yielded a fitted $K_{D}$ of 9.66 μM for doxorubicin and 7.97 μM for vancomycin in PBS (fig. S7), validating the platform’s ability to support reliable sensing of chemically and structurally distinct molecular targets via aptamer replacement. Moreover, both sensors on the BICEP shank demonstrated effective mass transport and reversible binding kinetics, evidenced by the rapid dual-sensor response stabilizing within the sampling interval of 3 min when sequentially challenged with 0, 40, and 0 μM vancomycin solution in PBS (fig. S8). These findings validate the biochemical sensing robustness and adaptability of the BICEP sensor platform.

We then evaluated the ability of the BICEP sensor to perform dual-compartment monitoring of dynamic changes in analyte concentration in real time. The kanamycin-targeting BICEP sensor shank was immersed in PBS, and aliquots of kanamycin stock solution were sequentially spiked in to induce concentration changes ranging from 0.01 to 10 mM. SWV was performed at one measurement per minute to interrogate both sensors, enabling real-time tracking of target binding and regeneration (Fig. 2C). Following this initial stepwise concentration challenge, we dip washed the device twice in kanamycin-free PBS and observed that the signal returned to baseline within one sampling cycle. Reexposure to 0.4 mM kanamycin confirmed sensor regeneration and yielded a reproducible measurement relative to the prior exposure to 0.4 mM kanamycin, after which we performed a final washout. Both blood and ISF sensors accurately tracked kanamycin concentrations, closely matching ground-truth values. We also performed dual-compartment sensing with the same BICEP shank at different SWV frequencies and observed reproducible kanamycin monitoring (fig. S9). The observed shifts in apparent sensitivity are attributable to frequency-dependent interrogation effects (fig. S10) (*23*, *30*). This dual-compartment, real-time sensing capability was similarly validated for vancomycin under the same protocol (fig. S11). Together, these results show that the BICEP platform offers a generalizable design for dual-compartment real-time molecular monitoring with high sensitivity and reversibility.

### BICEP enables wireless remote molecular monitoring

**Fig. 3.**
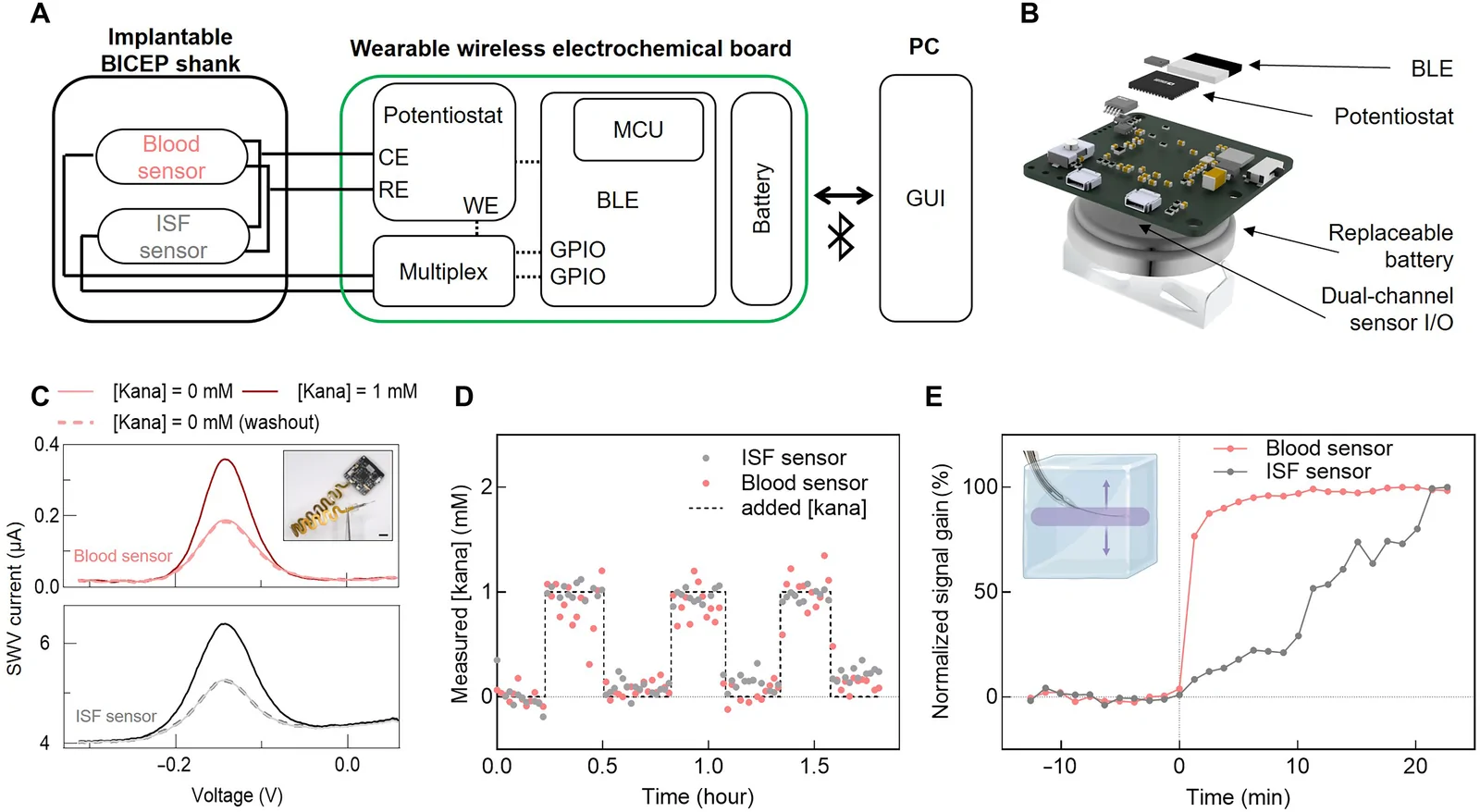
Wireless characterization of BICEP for dual-compartment, real-time molecular monitoring in vitro. (**A**) BICEP functional block diagram. CE, counter electrode; RE, reference electrode; WE, working electrode; GPIO, general-purpose input/output. (**B**) Exploded-view illustration of the wearable wireless electrochemical board, including BLE transmitter, potentiostat, battery, and sensor shank I/O. (**C**) Wirelessly acquired BICEP SWV data at 100 Hz from the blood (top) and ISF (bottom) sensors before spiking in kanamycin, after adding 1 mM kanamycin, and following washout with buffer. Inset shows the image of BICEP for wireless dual-channel SWV acquisition. Scale bar, 1 cm. (**D**) Wireless continuous monitoring with BICEP in undiluted human plasma, alternating between kanamycin-free and 1 mM kanamycin conditions. (**E**) Wireless dual-compartment monitoring with BICEP in vitro. The BICEP shank was inserted into a 1 wt % agarose phantom, with the blood sensor floating freely in the PBS-filled blood vessel–mimicking void channel and the ISF sensor embedded within the surrounding hydrogel, as shown in the inset. BICEP wirelessly monitors signals before and after infusion of a single 10 mM kanamycin bolus (marked by the dashed line) through the channel. Created in BioRender. X. Hou (2026), https://BioRender.com/l4ywcr1.

To enable the collection of measurements from awake, freely behaving animals, we coupled the sensor shank to a custom wireless communication unit, forming the complete BICEP system (Fig. 3A). As no existing system supports multiplexed SWV acquisition with a wearable form factor, we developed a compact, wireless potentiostat capable of rapid signal acquisition and remote real-time data transmission. This system incorporates a low-power electrochemical analog front-end potentiostat that performs multiplexed dual-channel voltammetry on demand (fig. S12); this potentiostat then relays the collected data to a PC or other device using a BLE module (Fig. 3B). The wearable unit is connected to the BICEP shank via an FPC, enabling consecutive SWV interrogation of the ISF and blood working electrodes. The configurable SWV parameters include the beginning potential, beginning equilibration time, ending potential, potential step, amplitude, and frequency, while the SWV readout includes voltages applied, forward and reverse currents, and SWV currents ($I_{\text{SWV}}=I_{\text{forward}}-I_{\text{reverse}}$). A tunable transimpedance amplifier circuit assists the potentiostat by amplifying the SWV readout currents (~0.01 to 10 μA) at the two sensing sites. The assembled wireless potentiostat is compact (29 mm by 29 mm by 7 mm), lightweight (8.9 g), and powered by a replaceable coin cell battery (CR2450) with an operational lifetime of over 1 month for a measurement interval of 1 min, making it well suited for monitoring freely moving rats (fig. S13). We calibrated our wireless potentiostat against a gold-standard electrochemical workstation using the same BICEP sensor and identical SWV conditions, and the overlapping voltammograms, together with highly reproducible kanamycin calibration curves (fig. S14), confirm that the wireless system preserves the analytical performance of the benchtop workstation. We also built a customized GUI to remotely configure the voltammetry settings and read the SWV data on the PC for real-time data analysis (fig. S15). The GUI can be used to configure the system for either single measurements or continuous measurements. After receiving the measurement request, the GUI compiles the dual-compartment SWV readout and the configuration as individual CSV files for downstream data analysis. We confirmed that when the BICEP system is active, the wireless unit can acquire voltammetry data from both the ISF and blood sensors on a single shank upon sequential exposure to kanamycin-free buffer, 1 mM kanamycin, and washout with buffer (Fig. 3C). The different SWV current scales for blood and ISF sensors arise from the different surface areas of the blood and ISF working electrodes. The acquired signals are automatically extracted, processed with custom MATLAB scripts, and visualized as time-lapse data, demonstrating real-time, remote molecular monitoring.

We validated BICEP’s robustness by assessing its mechanical resilience and functional stability in complex biological media. We subjected a BICEP shank to cyclic compressive strain (−25%), repeatedly bending and releasing it in PBS while wirelessly recording signals in both the bent and relaxed states. Both the blood and ISF sensors maintained SWV signal integrity with <3% variation across five compression-recovery cycles (fig. S16). To evaluate biochemical performance, we immersed the BICEP shank sequentially in pooled human plasma samples containing increasing and decreasing concentrations of kanamycin, with wireless signal acquisition every minute. Both sensors remotely tracked concentration changes between 0 and 1 mM within 1 min (Fig. 3D). We observed a small upward drift after each kanamycin exposure and dip-wash step, consistent with incomplete washing in the static plasma setup (fig. S17). In contrast, the signal remained stable within each kanamycin-free plasma segment, indicating that the nanostructured interface effectively suppressed sensor drift and supports rapid sensing and regeneration in protein-rich biological environments. Together, these results highlight BICEP’s ability to maintain mechanical robustness and operational stability under conditions that closely mimic in vivo sensing.

Last, we evaluated BICEP’s capacity to wirelessly monitor two distinct fluid compartments simultaneously in vitro. We developed an agarose hydrogel-based blood vessel phantom, where the blood sensor was inserted within a 1.7-mm-diameter fluidic channel, while the ISF sensor was embedded in the surrounding hydrogel (Fig. 3E). The channel was initially filled with PBS, which was then replaced with PBS containing 10 mM kanamycin under static conditions. Continuous SWV measurements were wirelessly sampled every minute. The blood sensor exhibited a rapid signal increase within 5 min following the introduction of 10 mM kanamycin into the channel, with the signal stabilizing shortly thereafter. In contrast, the ISF sensor recorded a gradual rise in signal over 25 min, reflecting delayed diffusion of kanamycin from the vessel into the initially analyte-free hydrogel. This distinct temporal response between the two sensors supports our hypothesis that BICEP can independently and wirelessly resolve analyte concentrations in both compartments, enabling real-time monitoring of intercompartment molecular transport.

### BICEP reveals kinetic differences in blood-ISF drug transport in anesthetized rats

**Fig. 4.**
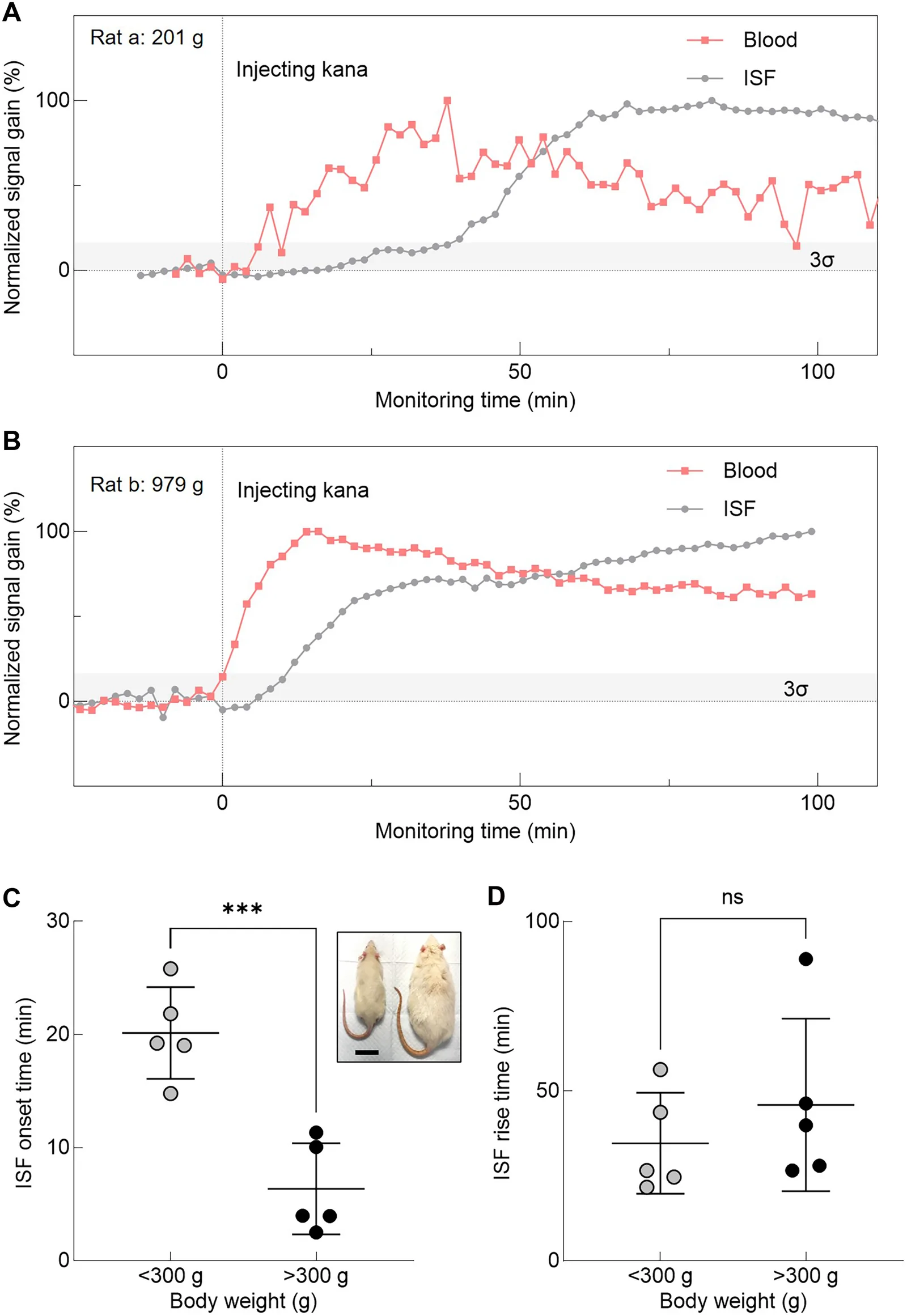
Continuous blood-ISF monitoring with BICEP sensors in anesthetized rats. (**A** and **B**) Continuous monitoring using the BICEP shank in (A) light (201 g) and (B) heavy (979 g) rats. The blood sensor was implanted in the femoral vein, with the ISF sensor interfacing with the fascia and interstitial space. A single bolus of kanamycin (40 mg/kg) was administered at the time marked by the dashed line. The gray-shaded region represents the ISF noise threshold, defined as three times the SD (3σ) of each sensor’s baseline signal before injection. (**C**) Scatter plot shows the relationship between body weight and ISF onset time after retro-orbital sinus injection of kanamycin (40 mg/kg) under anesthesia. Each dot represents an individual rat. Statistical analysis using Welch’s *t* test yielded a *P* value of 0.0007. The inset shows representative rats with varying body weights. Left: 199 g; right: 527 g. Scale bar, 5 cm. (**D**) Effect of body weight on ISF rise time. Statistical analysis using Welch’s *t* test yielded a *P* value of 0.4206. *n* = 10 rats for (C) and (D). ***P < 0.001; ns, not significant.

We subsequently evaluated the BICEP sensors for in vivo blood and ISF monitoring in anesthetized rats. We prepared the kanamycin BICEP sensor shank as described above, with a sterile suture wire threaded through two suture holes (diameter, 0.4 mm) positioned between the blood and ISF sections of the shank (fig. S18). After implanting the blood sensor into the rat’s femoral vein in the supine position, we used the suture to secure the shank to the vein, ensuring constant contact between the blood sensor and the bloodstream, while the ISF sensor remained in contact with the fascia throughout multihour anesthesia. Surgical closure secured the BICEP shank within the subcutaneous space and closed the wound region, with the FPC extended externally for potentiostat connection. Continuous monitoring was performed by connecting the BICEP shank’s FPC I/O to a multiplexed potentiostat for interleaved SWV interrogation. After establishing stable baselines (signal change < 2%) in both compartments for over 10 min, a single bolus of kanamycin (40 mg/kg) was administered to the rats via the retro-orbital sinus. For one of the rats (201 g), the blood sensor registered a rapid concentration increase upon the kanamycin injection, while the ISF sensor exhibited a delayed response, with an onset time of 25.8 min (Fig. 4A). We defined the onset time as the time point at which the ISF signal gain first exceeded the noise threshold, as calculated from the baseline signal of each sensor before injection (3σ). Although there are no prior reports of kanamycin onset delay in the ISF, previous studies on glucose—a similarly sized small molecule (<3 kDa)—have shown ISF delays of 0 to 45 min (average of ~25 min) relative to blood based on ISF-based continuous glucose monitoring and intermittent sampling in humans (*16*, *17*). These findings indicate that our system can capture delays in kanamycin transport from blood to ISF that were previously undetectable because of the lack of tools for monitoring both compartments simultaneously.

To further investigate interindividual differences in blood-ISF molecular transport, we repeated the same experimental protocol in a second rat with a substantially higher body weight (979 g) (Fig. 4B and fig. S19). Once again, blood kanamycin levels rose rapidly postinjection, reflecting the redistribution phase of pharmacokinetics, followed by a gradual decline during elimination. The ISF sensor also captured a delayed onset and distinct kinetic profile. Notably, the ISF onset time in this heavier rat was substantially shorter (10.05 min) than in the smaller animal. We therefore hypothesized that body weight may influence ISF transport kinetics and stratified animals into heavier (>300 g) and lighter (<300 g) groups (*31*) (*n* = 5 each). In this experiment, heavier rats exhibited significantly shorter ISF onset times (6.37 ± 4.03 min) compared to lighter rats (20.14 ± 4.05 min) (Fig. 4C, P = 0.0007). When we quantified the ISF rise time, defined as the period from ISF onset to peak, we observed no significant difference between the lighter (34.65 ± 14.88 min) and heavier (45.97 ± 25.39 min) groups (Fig. 4D).

To understand this phenomenon and building on prior studies of drug delivery from blood vessels to tumor tissue (*14*, *32*, *33*), we developed a blood-ISF mass transport model (Supplementary Text and figs. S1 and S2) and a phase diagram of ISF onset and rise times (fig. S3). When we overlaid our BICEP sensor in vivo data from rats of varying body weights onto this diagram, we observed shorter ISF onset times without a corresponding reduction in ISF rise times, a pattern consistent with enhanced diffusion in heavier rats. This is consistent with recent human studies reporting a positive correlation between body mass index and molecular diffusivity in various tissue microenvironments (*34*, *35*). Together, these results validate that BICEP sensors can accurately track blood-ISF molecular exchange kinetics continuously in real time and thereby reveal how individual physiological factors such as body weight influence intercompartmental transport.

### BICEP identifies distinct blood-ISF kinetics in freely moving versus anesthetized rats

**Fig. 5.**
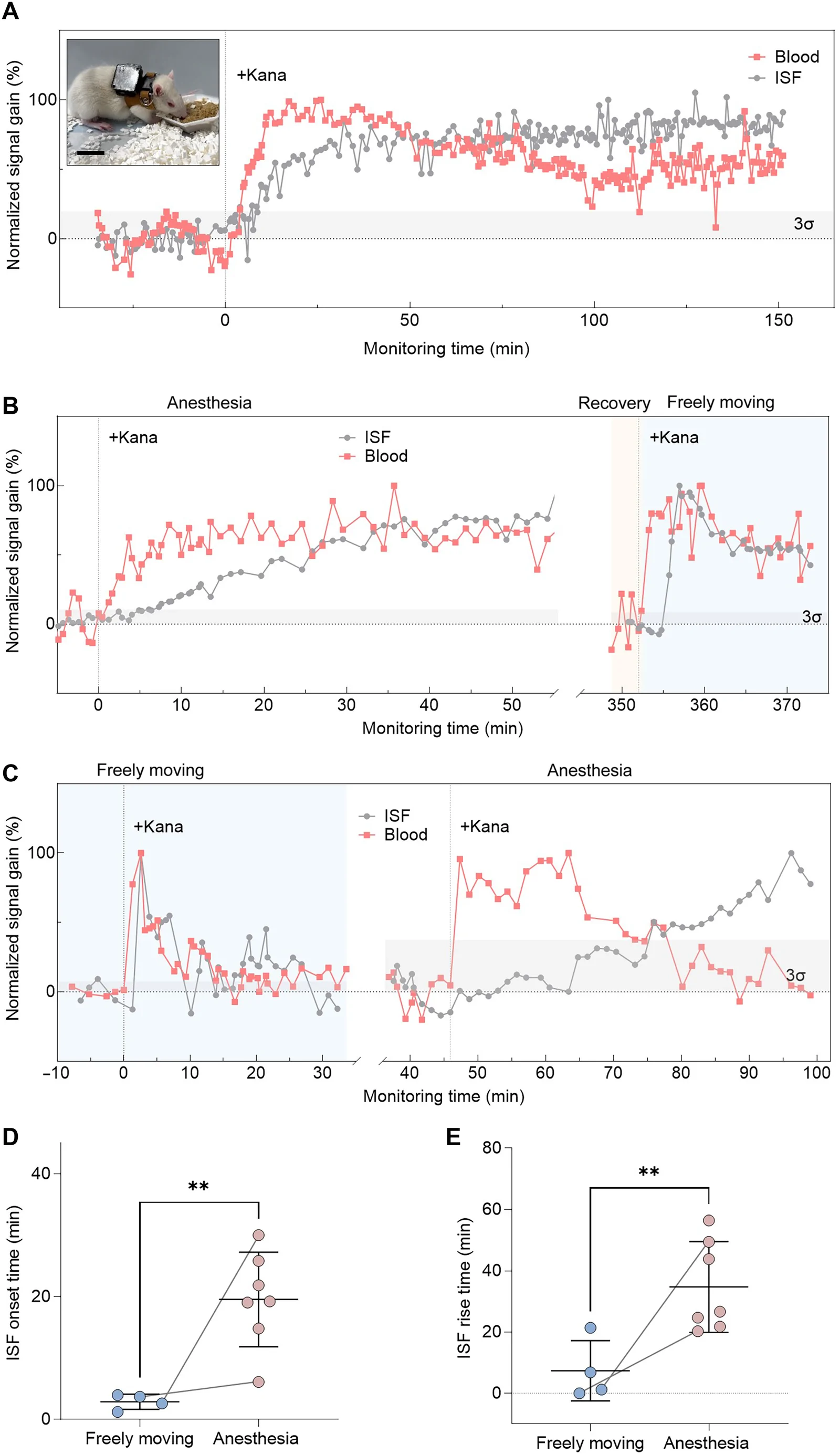
Wireless blood-ISF continuous monitoring in freely moving rats using the BICEP system. (**A**) Dual-compartment continuous kanamycin monitoring in blood and ISF of a freely moving rat using BICEP. A dose (40 mg/kg) of kanamycin was administered via retro-orbital injection during a 1-min period of isoflurane anesthesia (indicated by the dashed line). The rat (201 g) was acclimated to the wireless electrochemical board secured to a wearable jacket and implanted with the BICEP sensor shank, connected with a subcutaneously implanted stretchable FPC. The inset shows the rat performing natural eating behavior, demonstrating the system’s compatibility with unrestrained physical activity. The gray-shaded region represents the ISF noise threshold, defined as three times the SD (3σ) of each sensor’s baseline signal before injection. Scale bar, 3 cm. (**B** and **C**) Wireless, continuous blood-ISF monitoring across anesthetized and freely moving conditions in two individual rats. (B) Two doses (40 mg/kg) of kanamycin were administered in total: the first dose under anesthesia, followed by a second dose postrecovery in the freely moving animal. (C) In another rat, two doses (40 mg/kg) of kanamycin were administered in total: the first dose during the freely moving stage, followed by a second dose under anesthesia. Injection time points are indicated by dashed lines. In both (B) and (C), signal traces are independently normalized to the peak response following each injection to highlight the dynamic differences in molecular kinetics between physiological states. (**D** and **E**) Impact of consciousness on ISF onset time (D) and ISF rise time (E). Each dot represents an individual rat, with connecting lines indicating transitions of the same rat between states. Statistical analysis using Welch’s *t* test yielded *P* = 0.0011 for (D) and *P* = 0.0055 for (E). **P < 0.01.

Last, we demonstrated that BICEP enables simultaneous monitoring of both blood and ISF in awake, freely moving animals. For these experiments, the wireless unit was enclosed in a compact 3D printed case (3 cm by 3 cm by 1 cm; fig. S20) and mounted onto a commercial rodent vest to ensure comfortable wear. The BICEP sensor shank was implanted into the femoral vein and interstitial space under anesthesia as detailed above and reinforced with an additional stiffening layer to enhance mechanical stability during motion. To minimize motion-induced artifacts, we anchored the shank in the interstitial space using predesigned suture holes, aligned with anatomical landmarks for secure fixation. A custom-designed serpentine FPC was implanted subcutaneously to connect the wearable unit to the implanted sensor. The serpentine geometry was specifically engineered to enhance stretchability and relieve mechanical strain during movement, preventing stress transfer to the sensing interface (fig. S21). Following wound closure and cessation of anesthesia, rats typically recovered within 1 to 10 min and exhibited normal behavior including running, grooming, and eating (Fig. 5A and movies S1 to S3). Over the course of our experiments, we wirelessly transmitted SWV readouts from both blood and ISF sensors on the implanted BICEP shank to a Bluetooth-connected laptop, where they were automatically analyzed and visualized as time-lapse traces in real time (fig. S22 and movie S1).

We started wireless blood-ISF monitoring on one freely moving rat (201 g). Its blood kanamycin levels peaked within 10 min, followed by a gradual decline over several hours, after a single-bolus injection of kanamycin (40 mg/kg; Fig. 5A). Notably, this freely moving rat exhibited a brief ISF onset delay of 3.95 min, which is significantly shorter than what we observed in the cohort of lightweight anesthetized rats shown in Fig. 4C (20.14 ± 4.05 min; *P* < 0.001, *Z* = −4.00). We hypothesized that the accelerated blood-ISF interplay was influenced by consciousness. To experimentally validate the distinct blood-ISF kinetics between freely moving and anesthetized states, we performed longitudinal dual-compartment monitoring in the same animals under both conditions. In one representative rat (268 g; Fig. 5B), identical kanamycin doses (40 mg/kg) were administered first under anesthesia and later shortly before transitioning to the freely moving state. Both blood and ISF exhibited markedly faster kinetics in the conscious state: The blood peak time decreased from 35.70 to 7.62 min, and the ISF profile showed a shorter onset delay (3.70 versus 6.08 min) with a kinetic trajectory that closely mirrored the blood dynamics, with a rapid ISF rise time of 1.23 min. We observed similar patterns in a second rat when the sequence of freely moving and anesthetized conditions was reversed (Fig. 5C).

Replication of blood-ISF monitoring with BICEP in other freely moving lightweight rats yielded comparable results, with markedly shorter ISF onset delays (2.87 ± 1.24 min) compared to anesthetized lightweight rats (*P* = 0.0011; Fig. 5D). Moreover, BICEP monitoring revealed significantly faster ISF rise times in freely moving lightweight rats (7.38 ± 9.82 min) compared with their anesthetized counterparts (*P* = 0.0055; Fig. 5E). Phase diagram analysis of BICEP measurements confirmed enhanced blood-ISF convection in freely moving subjects (fig. S3D). These findings are consistent with reports of increased blood flow, elevated metabolic rates, and restored cardiovascular dynamics in awake versus anesthetized states (*36*–*38*), highlighting the importance of physiological context when interpreting ISF measurements. BICEP’s simultaneous dual-compartment monitoring in freely moving subjects thus makes it possible to accurately link ISF signals to blood analyte levels across diverse physiological conditions.

## DISCUSSION

In this work, we present BICEP—a platform that can continuously and simultaneously measure small-molecule drugs in blood and ISF and thereby directly quantify in vivo transport between the two compartments. Our platform combines microfabricated probes featuring nanostructured antifouling interfaces and reversible aptamer switches for stable in vivo operation, a custom wearable potentiostat for wireless SWV acquisition, and an implantation workflow that enables continuous monitoring in freely moving subjects. To the best of our knowledge, this represents the first demonstration of continuous monitoring of biochemical dynamics in both blood and ISF of freely moving subjects in real time. BICEP enables direct measurement of dynamic blood-ISF interplay, offering previously inaccessible insights into physiological transport and pharmacokinetics at the individual level and thereby revealing how transit between these two fluid compartments is influenced by both cross-sectional factors such as body weight and longitudinal variables such as the transition between anesthetized and conscious states. Our findings further demonstrate the importance of continuous dual-compartment monitoring to avoid oversimplified interpretations of ISF data and establish appropriate parameters for the real-world diagnostic utility of such data.

While these results represent a substantial advance toward real-time, multicompartment biochemical monitoring in individual subjects, there are several avenues for the further development and optimization of our platform. First, we anticipate that emerging minimally invasive blood and ISF sampling methods will enable more extensive cross-validation of BICEP-derived blood and ISF target concentrations. For blood, we have demonstrated close agreement between continuous BICEP monitoring and episodic blood sampling under sedation, followed by enzyme-linked immunosorbent assay (ELISA), which yielded similar concentration-time profiles (fig. S23). For ISF, however, rapid and repeated sampling at sufficient volume remains challenging (*2*), and current techniques limit time-resolved validation without perturbing local tissue and systemic physiology. Second, we are actively broadening the range of monitorable molecular targets. We are pursuing rational design and high-throughput screening strategies to develop reversible switches capable of detecting a wider range of macromolecules, including peptide hormones, cytokines, and other clinically relevant protein biomarkers (*39*–*42*). Third, achieving finer spatiotemporal resolution with increased sensing sites and faster sampling rates would enable the tracking of rapid biochemical exchange processes, such as neurotransmitter signaling within spatially confined regions. We also see opportunities to engineer a miniaturized multichannel potentiostat with ultralow power consumption to facilitate simultaneous monitoring across multiple compartments with enhanced precision (*43*). Last, to advance the translational value of this platform, we are looking into deploying BICEP in larger animal models to characterize blood-ISF transport dynamics under physiologically relevant conditions, assess procedural safety, and inform the design of subsequent human studies. Collectively, these ongoing efforts should help enable the BICEP platform to accelerate the transition from invasive blood-based diagnostics to minimally invasive, wearable-compatible ISF monitoring for a broad spectrum of analytes.

## MATERIALS AND METHODS

### Materials

All chemicals were purchased from Sigma-Aldrich unless otherwise noted. Thiolated branched PEG (8-arm PEG-SH; molecular weight, 20,000), was ordered from Creative PEGWorks. Pooled human plasma (blood-derived) was purchased from Innovative Research. Nuclease-free water (NFW) was purchased from Thermo Fisher Scientific. PBS buffer was prepared by diluting 10× PBS and MgCl_2_ with NFW to 1× PBS and 2 mM Mg^2+^. Kanamycin monosulfate (USP grade) was purchased from Gold Biotechnology. 3D printing resin (clear) was purchased from Anycubic. Polydimethylsiloxane (PDMS; SYLGARD 184) was purchased from Dow. FFCs, digital multimeter, ecoflex, glue, vetbond, nonabsorbable suture (USP 4-0), and rodent vests were purchased from Amazon. Customized FPCs were purchased from PCBWay. The board was assembled by Conclusive Engineering. Kanamycin ELISA Kit (MET-5144) was purchased from Cell Biolabs Inc. The aptamer switches were ordered from Integrated DNA Technologies. Sequences used were as follows: kanamycin aptamer, /5ThioMC6-D/GGG ACT TGG TTT AGG TAA TGA GTC CC/3MeBlN/; doxorubicin aptamer, /5ThioMC6-D/ ACC ATC TGT GTA AGG GGT AAG GGG TGG T /3MeBlN/; vancomycin aptamer, /5ThioMC6-D/CGA GGG TAC CGC AAT AGT ACT TAT TGT TCG CCT ATT GTG GGT CGG/3MeBIN/. 5ThioMC6-D indicates a thiol modification at the 5′ end, and 3MeBlN indicates methylene blue labeling at the 3′ end.

### Fabrication of BICEP microelectrode array sensor

A schematic of the device fabrication process is shown in fig. S5. To begin, a 200-nm-thick Al sacrificial layer was deposited on a 4-inch (10.16-cm) Si wafer by electron beam evaporation (ATC-E, AJA International). To ensure robust SU8 adhesion onto the Al layer, a hexamethyldisilazane adhesion promoter layer was coated on the Al layer before SU8 coating in a vapor prime oven (Yield Engineering Systems). A 30-μm-thick SU8 layer (SU8 3025, Kayaku Advanced Materials) was then spin coated onto the Al layer and thermally baked at 95°C for 12 min. The SU8 probe substrate was patterned via photolithography using a contact mask aligner (Karl Suss MA6, SÜSS MicroTec) and then hard baked at 160°C for 15 min on a hot plate. Subsequently, a 150-nm-thick SiO_2_ protective layer was deposited onto the probe by electron beam evaporation to protect the SU8 layer from damage inflicted by nitric acid in the nanoporous etching step.

For the nanoporous electrode fabrication, a 4.5-μm-thick positive photoresist layer (MEGAPOSIT SPR220-3, Kayaku Advanced Materials) was spin coated onto the wafer and then patterned using maskless photolithography (MLA150, Heidelberg Instruments). A Ti/Au (10/90 nm) bottom layer was deposited via sputter deposition (LAB Line SPUTTER, Kurt J. Lesker Co.). A 300-nm-thick Au-Ag alloy layer (66.7% Ag/33.3% Au) was then deposited by cosputtering Au and Ag. The wafer was then immersed in 69% (v/v) nitric acid for 7 min to dissolve the Ag, forming a nanoporous Au layer. A lift-off process was then performed in acetone to form the gold nanoporous electrodes.

Last, the probe was passivated with a 9-μm-thick SU8 encapsulation layer (SU8 3005, Kayaku Advanced Materials), leaving the electrode area open. The sensor was released from the wafer through anodic dissolution of the Al sacrificial layer. For this step, the Si wafer was immersed in 2 M NaCl, and 15-V dc voltage was applied to the Al layer. The reference electrode was made by electroplating an Ag layer onto the electrode region via 45-s chronoamperometry at −0.8 V in 50 mM potassium dicyanoargentate solution, followed by a 10-min etching procedure in bleach solution. Electroplating of the sensor shank reference electrode was performed using a 50 mM potassium dicyanoargentate solution in 0.25 M sodium carbonate with external Ag/AgCl and Pt electrodes, applying chronoamperometry at −0.8 V for 45 s. The microelectrode array sensor shank was stored in dark desiccator before usage.

### Assembly of BICEP sensor shank

The microelectrode array sensor was connected to an FFC or FPC using anisotropic conductive film, secured by a customized 3D-printed connector and 10:1 PDMS (fig. S6). Successful electrical connections were confirmed by low contact resistance (<10 Ω) without shorting to adjacent pins, as measured with a multimeter (Fluke-15B+, DigiKey). The assembled BICEP sensor shanks, consisting of the sensor shank and cable, were incubated in a 67°C oven for 30 min for PDMS curing. The shanks were washed with 70% isopropyl alcohol and NFW before aptamer switch immobilization.

### BICEP sensor preparation and functionalization

Aptamer switches were prepared at a final concentration of 100 μM in NFW and stored at −20°C until use. To expose free thiol groups for subsequent immobilization, a 1000-fold molar excess of freshly prepared tris(2-carboxyethyl)phosphine hydrochloride was added to the aptamer solution and incubated for 40 min at room temperature in the dark. The reduced aptamer solution was then diluted to 1 μM in PBS. PEG solution was prepared by dissolving 1 wt % in 70% isopropyl alcohol at 65°C, followed by sonication. Polymer coating was then performed by incubating the aptamer-functionalized BICEP sensor shanks with this PEG solution at room temperature for 3 hours. The sensor shanks were then rinsed twice with excess PBS and incubated with ~10 mM MCH for 3 hours at room temperature to fully passivate the remaining exposed gold surface. Last, the BICEP sensor shanks were washed twice with excess PBS and stored in PBS at room temperature before electrochemical measurements.

### Electrochemical characterization

Electrochemical measurements were performed using a PalmSens 4 potentiostat (PalmSens), an EmStat4 potentiostat with multiplexer (MUX8-R2 or MUX8, PalmSens), or the wearable wireless electrochemical board. SWV was conducted over a potential range of approximately −0.5 to 0.0 V, with an amplitude of 20 mV, a step size of 1 mV, and pulse frequencies ranging from 50 to 200 Hz. Dose-response curves were obtained by exposing the sensor shanks to target analyte concentrations either through spiking the medium with target stock solution (final dilution < 1%, v/v) at room temperature or by immersing the sensor directly in premixed media containing the desired analyte concentrations. For electrochemical characterization using a commercial potentiostat, SWV data were processed and visualized in real time using custom MATLAB scripts (MATLAB R2023a), which automatically extracted SWV peak current levels. For measurements using the wireless system, a custom-designed GUI was used to wirelessly configure SWV parameters and sampling rates, as well as to extract SWV data. The resulting SWV data files were automatically detected, validated for complete waveform acquisition, and analyzed in MATLAB to extract peak current levels, which were continuously plotted as time-lapse traces for individual channels. Signal gains were normalized to the maximum value within the time range unless otherwise specified. Calibration curves were generated by fitting the dose-response data to the Langmuir isotherm model using Excel 365 and GraphPad Prism 10.

### Surface and tomography characterization by scanning electron microscopy

For scanning electron microscopy (SEM) characterization, samples were mounted on pin stubs using conductive silver paint (Ted Pella), followed by sputter coating (Leica EM ACE600 sputter coater) with a 4-nm gold layer to enhance conductivity. Sensor imaging was performed using SEM systems including the Thermo Fisher Scientific Apreo S LoVac, FEI Magellan 400 XHR, and FEI Helios NanoLab 600i DualBeam. Before imaging, samples underwent plasma cleaning within the SEM chamber. Imaging was conducted at accelerating voltages of 2 to 5 keV. Analysis began with wide-field imaging to survey the overall structure, followed by high-resolution imaging of at least four randomly selected regions for detailed assessment.

### Live animal studies

Live animal studies were conducted using male Sprague-Dawley rats under Stanford Administrative Panel on Laboratory Animal Care protocol #33226. All rats were obtained from Charles River Laboratories, with body weights ranging from 150 to 1000 g and ages 1 to 6 months. Anesthesia was induced and maintained with 1 to 2.5% isoflurane gas during all surgical procedures, and the animals were continuously monitored throughout. Surgical sites were clipped and disinfected using three alternating rounds of betadine surgical scrub and alcohol. Rats were placed on a rodent heating pad during the procedures, and eye lubricant was applied to prevent corneal drying.

For continuous comonitoring under anesthesia, the femoral vein was surgically exposed through a small incision. The blood sensor segment of the BICEP shank was inserted and advanced into the vein, while the ISF sensor segment was positioned to face the fascia above the vein, with the animal in a supine position. The shanks were secured in place using prefixed suture wires wrapped around the femoral vein between the blood and ISF sensors. Surrounding fat tissue was sutured over the ISF sensor to maintain tight contact with the interstitial space. The FFC or FPC cable was routed externally through the wound and connected directly to either a commercial potentiostat or the customized wireless system for real-time dual-compartment monitoring. In the animal experiments, the decision to administer kanamycin was based on real-time analysis of the raw SWV peak values. As illustrated in fig. S19, we continuously extracted SWV peak amplitudes over time and then computed a running average. Once the baseline variation was <2% over a 10-min window relative to this running average, kanamycin was administrated via retro-orbital sinus injection.

For continuous dual-compartment monitoring in freely moving animals, rats were first acclimated to a properly fitted rodent vest. Following surgery, the animals were anesthetized and fitted with a vest housing the wireless system. Long-lasting analgesia was provided via subcutaneous injection of Ethiqa XR and Carprofen. A customized stretchable FPC with serpentine design (fig. S21) connected the BICEP sensor shank to the wireless system through a 3D printed connector, designed to relieve mechanical strain during movement and maintain shank stability. Two incisions were made: one near the back edge of the vest and another near the hind limb. The stretchable FPC and attached BICEP sensor shank were tunneled subcutaneously between these incisions. Implantation followed the same procedure as in the anesthetized setup, with additional sutures around the ISF sensor to minimize relative motion against interfacing tissues. The leg incision was then closed, and the stretchable FPC was routed through the back incision and connected to the wireless electrochemical module before waking the animal on a heating pad. Rats could move freely and behave naturally in their individual home cages, with wet food provided. Any signs of abnormal movement were addressed immediately. After baseline monitoring in the absence of kanamycin, a 100 mM kanamycin solution was administered via retro-orbital sinus injection at a dose of 40 mg/kg under brief anesthesia. At the end of the experiment, animals were euthanized by exsanguination under general anesthesia.

Blood kanamycin ELISA quantification was performed by collecting 100 to 250 μl of blood from the rat tail vein into lithium heparin Microtainer tubes under anesthesia, with the sampling period recorded for each draw. The samples were centrifuged at 10,000 rcf for 10 min, and the resulting plasma was stored at −20°C until analysis with a kanamycin ELISA kit.

### Statistics and reproducibility

All experiments were repeated at least three times. Data are shown as means ± SD (*n* ≥ 3) and were analyzed with GraphPad Prism 10 software. Differences are considered statistically significant at *P* < 0.05.
