# Supplementary material for: Continuous monitoring of blood–interstitial fluid intercompartmental molecular kinetics in freely moving animals
Source: Sci Adv. 2026 May 20;12(21):eaed1895. doi: 10.1126/sciadv.aed1895 (PMC13189097; doi:10.1126/sciadv.aed1895)
Supplement: Supplementary file 1 [file sciadv.aed1895_sm.pdf]

Science Advances
AAAS

Supplementary Materials for

Continuous monitoring of blood–interstitial fluid intercompartmental molecular kinetics in freely moving animals

Yihang Chen *et al.*

Corresponding author: Yihang Chen, yihangch@stanford.edu; Hyongsok Tom Soh, tsoh@stanford.edu

Sci. Adv. **12**, eaed1895 (2026)
DOI: 10.1126/sciadv.aed1895

The PDF file includes:

Supplementary Text
Figs. S1 to S23
Legends for movies S1 to S3

Other Supplementary Material for this manuscript includes the following:

Movies S1 to S3

Supplementary Text

<u>Blood-ISF transport model</u>

Spatiotemporal distribution of molecules following retro-orbital injection reflects the dynamic transport and clearance processes within the body, as shown below:

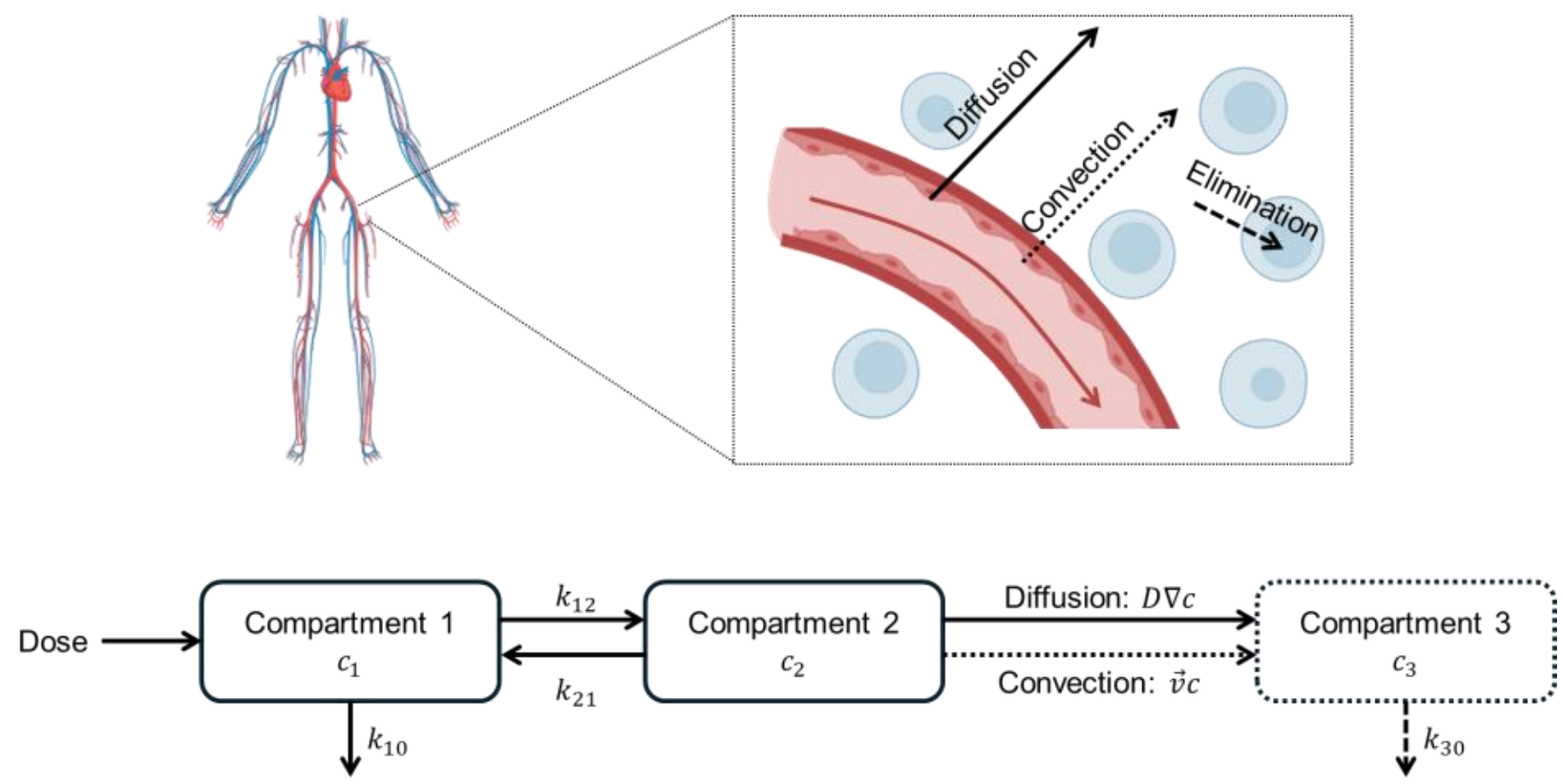

Fig. S1. Schematic of blood-ISF transport model. Created in BioRender. hou, X. (2026) https://BioRender.com/l4ywcr1.

The administered molecules initially mix within the central compartment (compartment 1) and subsequently exchange with the circulating blood (compartment 2), where the blood sensor is interfaced. This phase can be modeled using a two-compartment pharmacokinetic framework, described by the following system of ordinary differential equations:

$$\frac{dc_1}{dt} = -k_{10}c_1 - k_{12}c_1 + k_{21}c_2 \qquad (S1)$$

$$\frac{dc_2}{dt} = k_{12}c_1 - k_{21}c_2 \qquad (S2)$$

Molecules are then transported from the bloodstream into the interstitial space (compartment 3), where the ISF sensor resides. This transport involves both diffusion and convection mechanisms, while concurrent consumption or elimination may occur within the interstitial compartment, based

on prior studies of drug delivery from blood vessels to tumor tissue. Given that the ISF sensing volume is much smaller than that of the blood sensor, we assume that ISF transport exerts minimal influence on upstream blood concentrations. To capture the spatiotemporal evolution of molecular concentrations within the ISF, we use a partial differential equation model that includes terms for convection, diffusion, and elimination:

$$\frac{\partial c_3}{\partial t} = -v\frac{\partial c_3}{\partial x} + D\frac{\partial^2 c_3}{\partial x^2} - k_{30}c_3 \qquad (S3)$$

The boundary and initial conditions for this model are defined as follows:

$$c_3(x=0,t) = c_2(t) \qquad (S4)$$

$$\left.\frac{\partial c_3}{\partial x}\right|_{distal} = 0 \qquad (S5)$$

$$c_3(x,t=0) = 0 \qquad (S6)$$

Model simulations demonstrate that modulating convection, diffusion, and elimination dynamics meaningfully shapes the spatiotemporal transport of molecules from blood to ISF, as shown below:

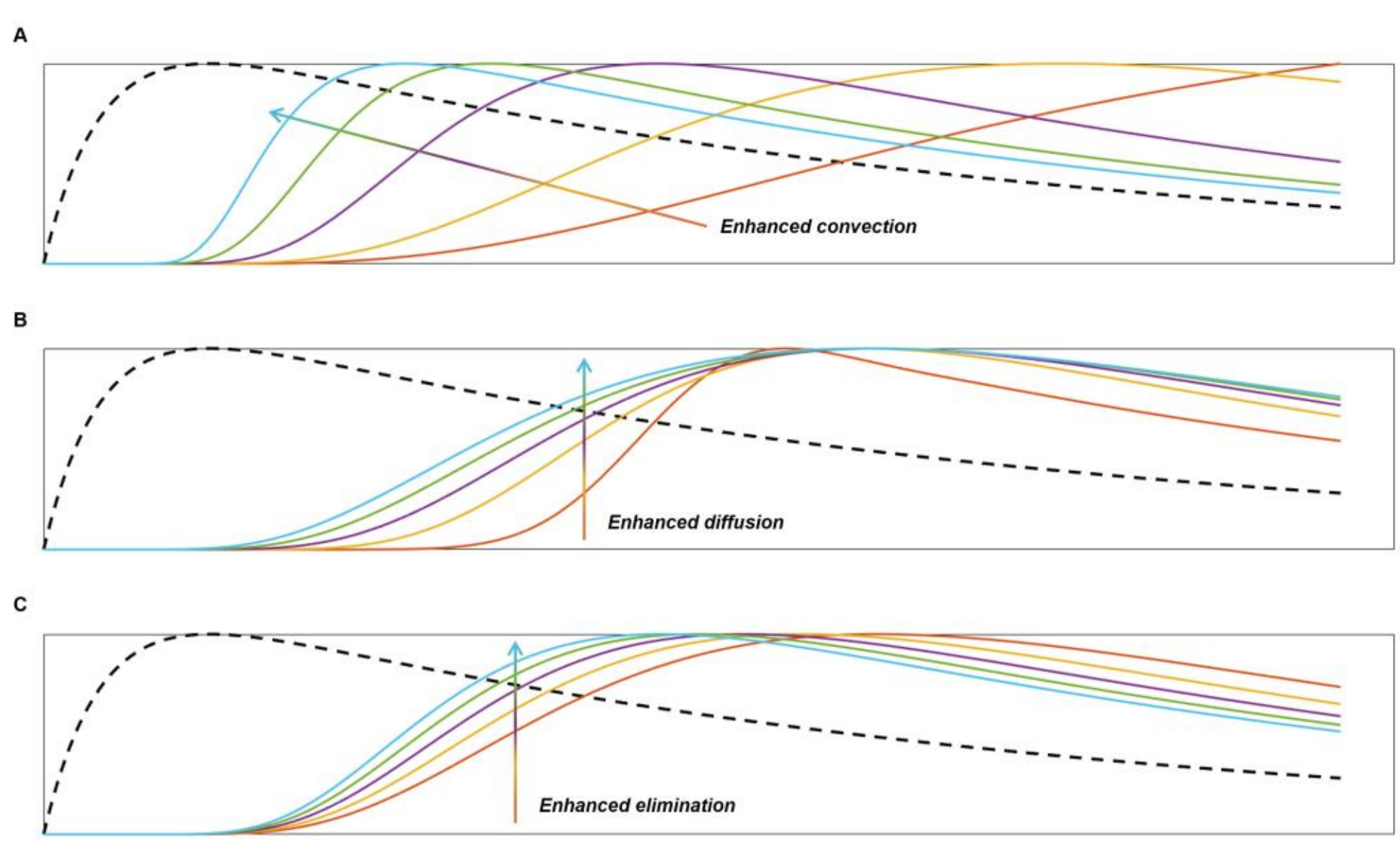

Fig. S2. Blood-ISF transport simulations. Modulating convection (**A**), diffusion (**B**), and elimination (**C**) dynamics meaningfully shapes the spatiotemporal transport of molecules from blood to ISF.

Increasing convection from blood to ISF accelerates molecular transport, resulting in ISF concentration profiles that more closely reflect those of blood. Under these conditions, the peak ISF concentration occurs earlier. In contrast, enhancing diffusion extends the ISF rise time.

We defined the simulated ISF onset time as the point when ISF signal crosses a 3% normalized threshold, and the simulated ISF rise time as the time from ISF onset to signal peak. We then defined a phase diagram of ISF onset and rise times, visualizing the convection, diffusion, and elimination effects to blood-ISF interplay, as shown below:

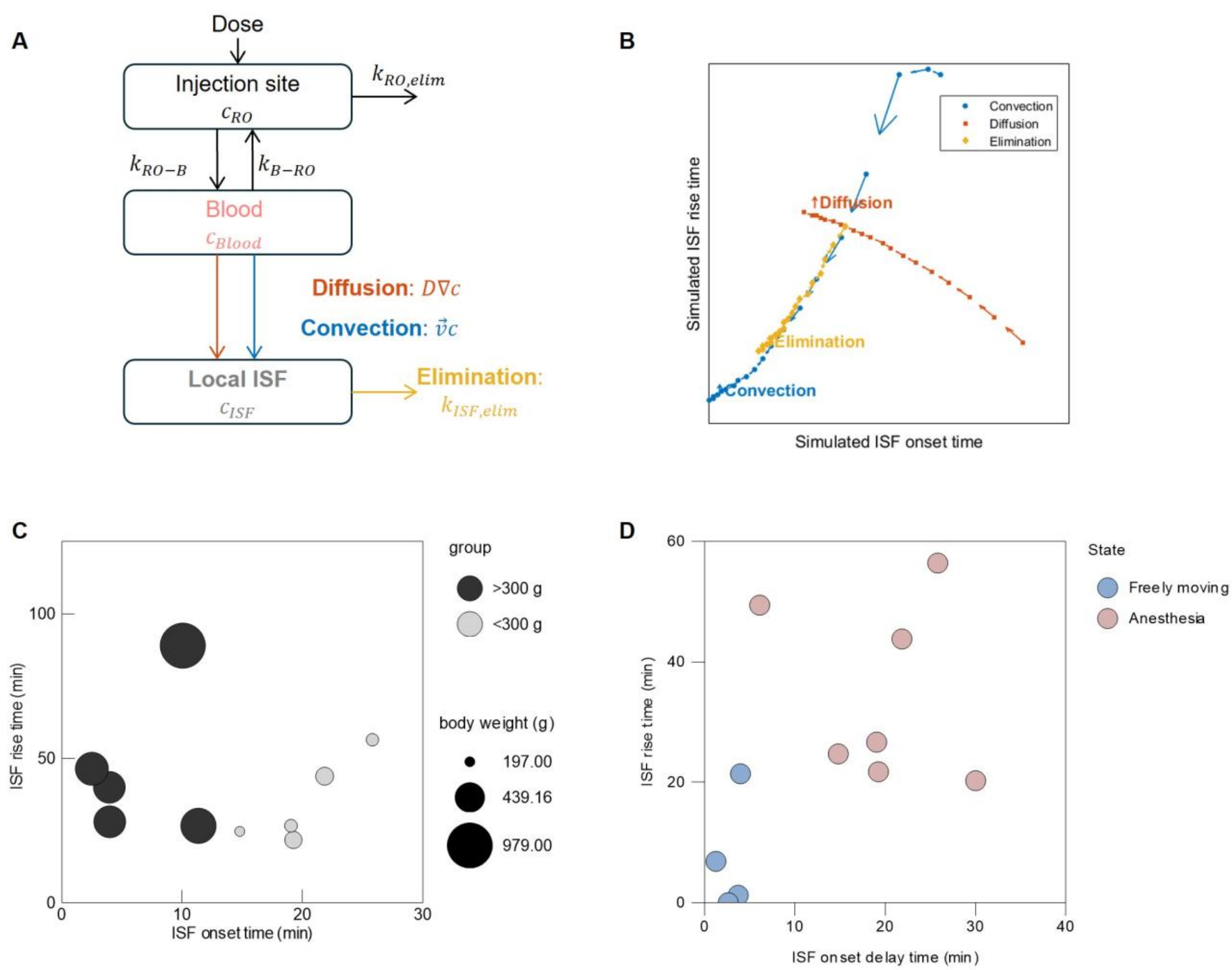

Fig. S3. Phase diagram of ISF onset and rise times visualizing blood-ISF interplay. (**A**) The proposed blood-ISF transport model. (**B**) Phase diagram depicting the distinct impacts of diffusion, convection, and elimination on ISF onset and rise times. (**C**) Phase diagram of ISF onset and rise times measured by the BICEP shank, stratified by body weight. Dot size represents individual rat body weight. (**D**) Phase diagram of ISF onset and rise times measured by wireless BICEP, stratified by consciousness state.

Collectively, these transport and clearance mechanisms govern the temporal distribution of molecules between compartments and shape the dynamic trajectory of ISF signals.

Additional Figures

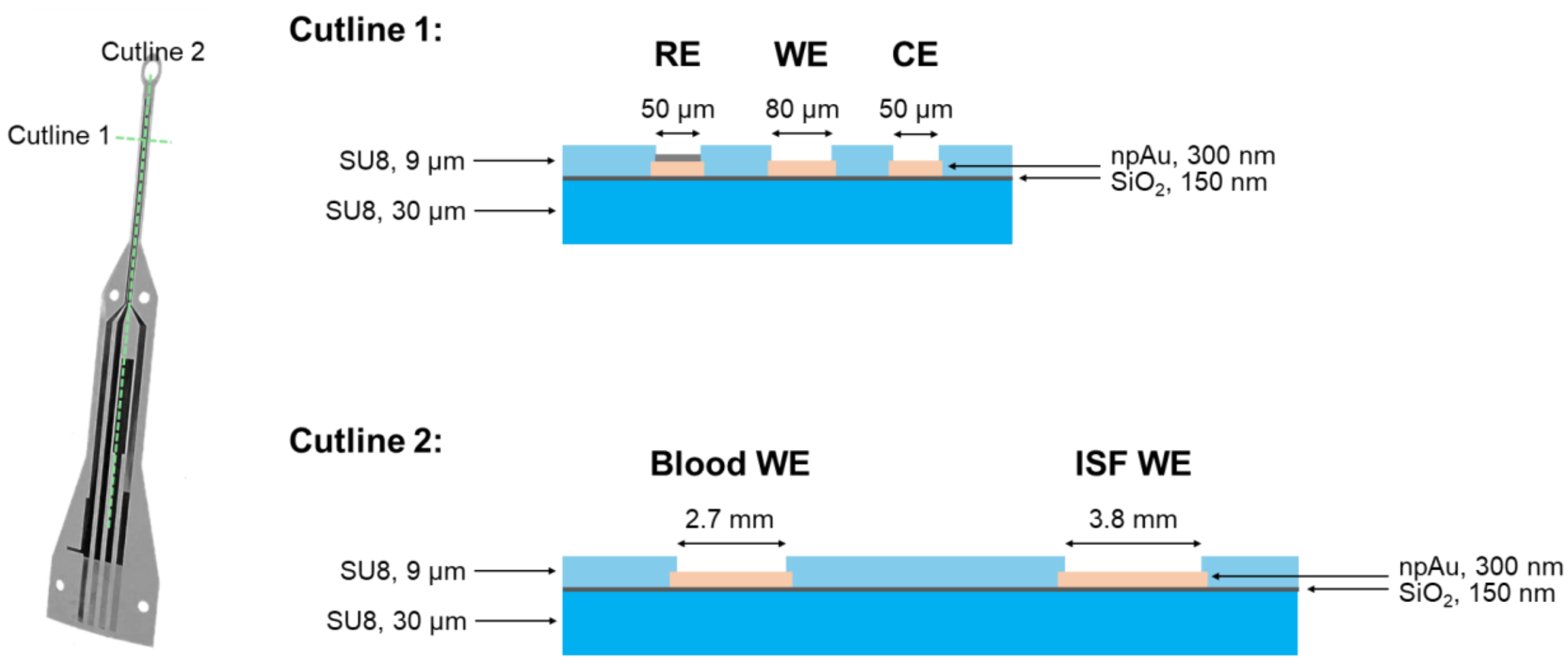

Fig. S4. Geometric design of the BICEP sensor shank. Schematic and cross-sectional illustration of the BICEP shank, showing the spatial arrangement of the blood- and ISF-sensing working electrodes (WE), along with the reference (RE) and counter electrodes (CE).

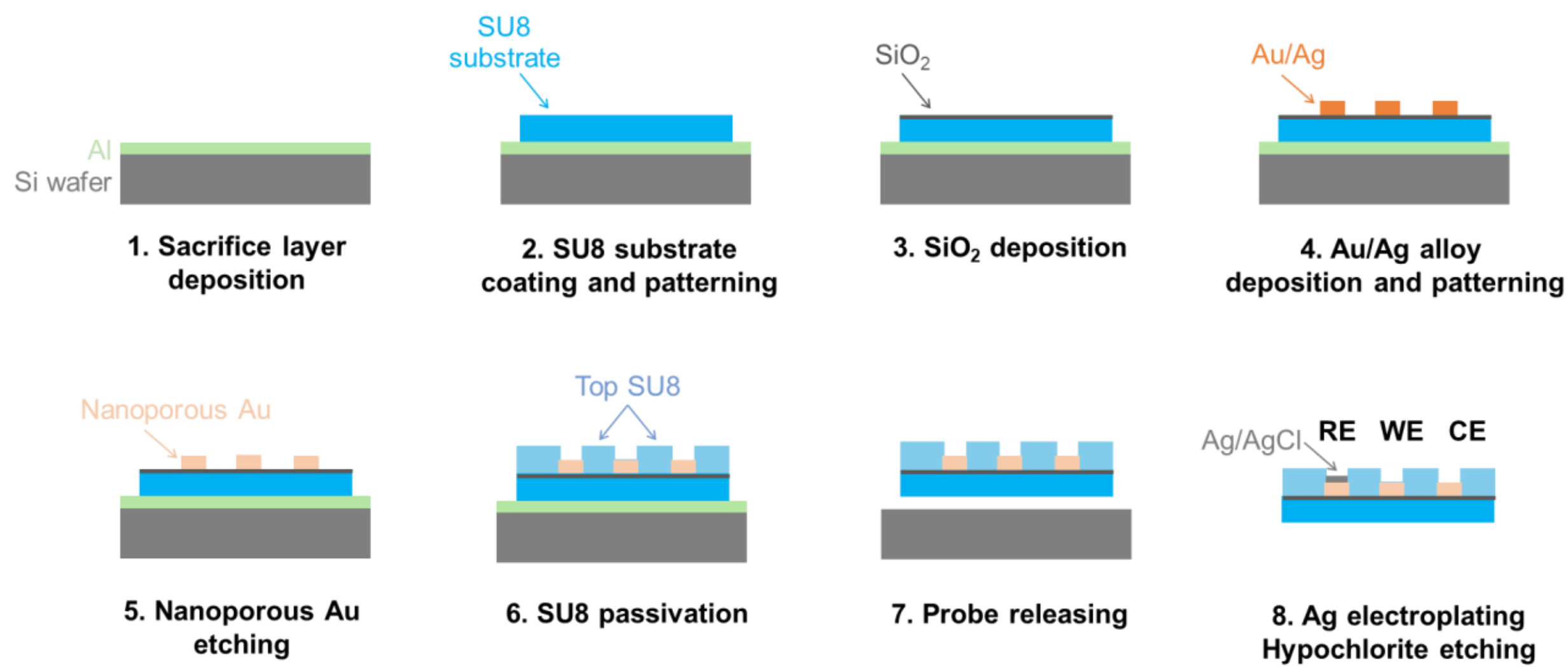

Fig. S5. Fabrication workflow for the BICEP sensor shank. The BICEP sensor shank fabrication workflow is detailed in full in the Methods section.

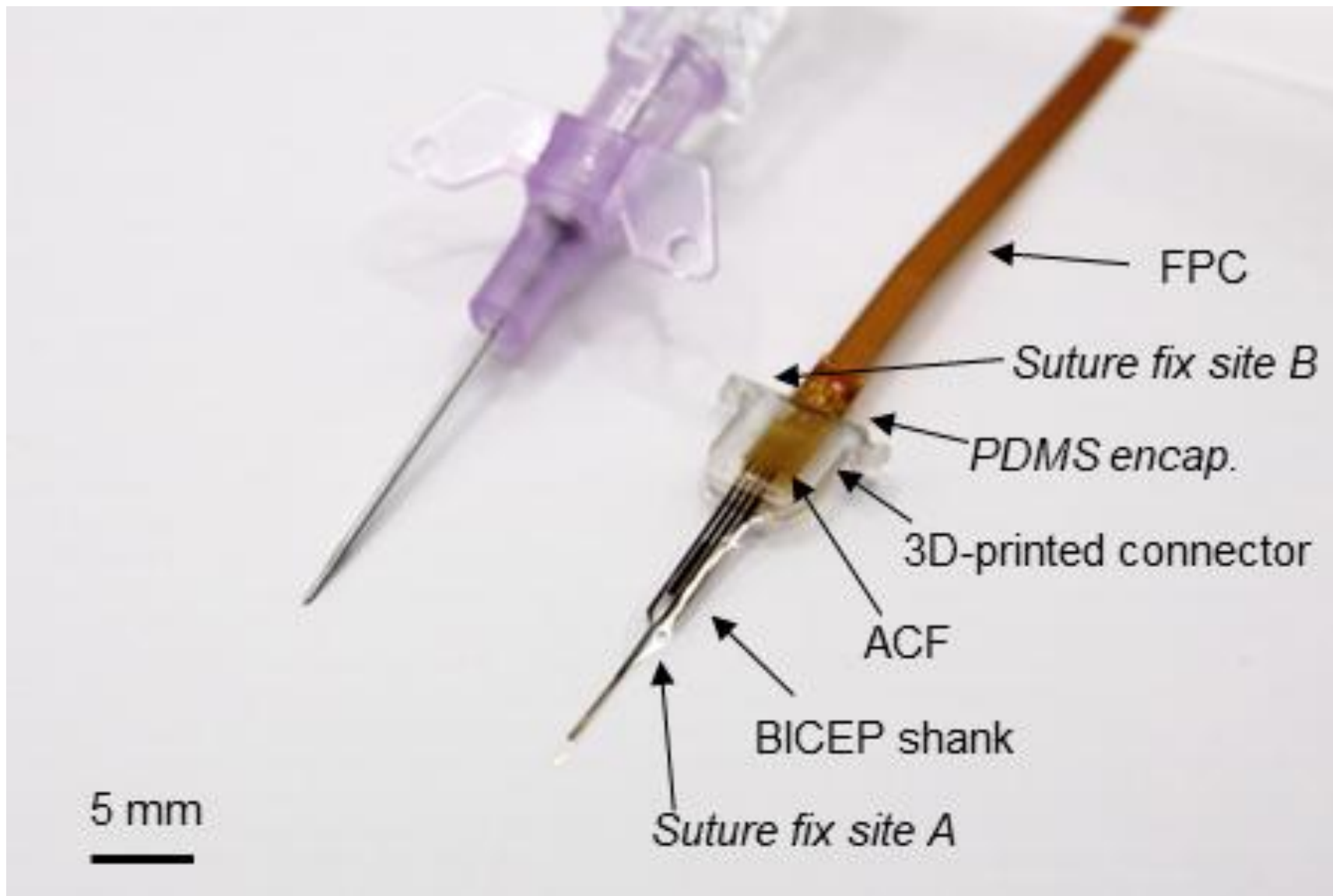

Fig. S6. Image of the BICEP shank with integrated blood and ISF sensors alongside a 26G IV catheter. The BICEP shank blood sensing region is smaller than a 26G IV catheter, which is suitable for peripheral venous implantation. The BICEP shank is assembled with the flexible printed cable (FPC) via an anisotropic conductive film (ACF). To further secure the electrical connection, the junction is mechanically supported by a 3D-printed connector, which is glued with PDMS.

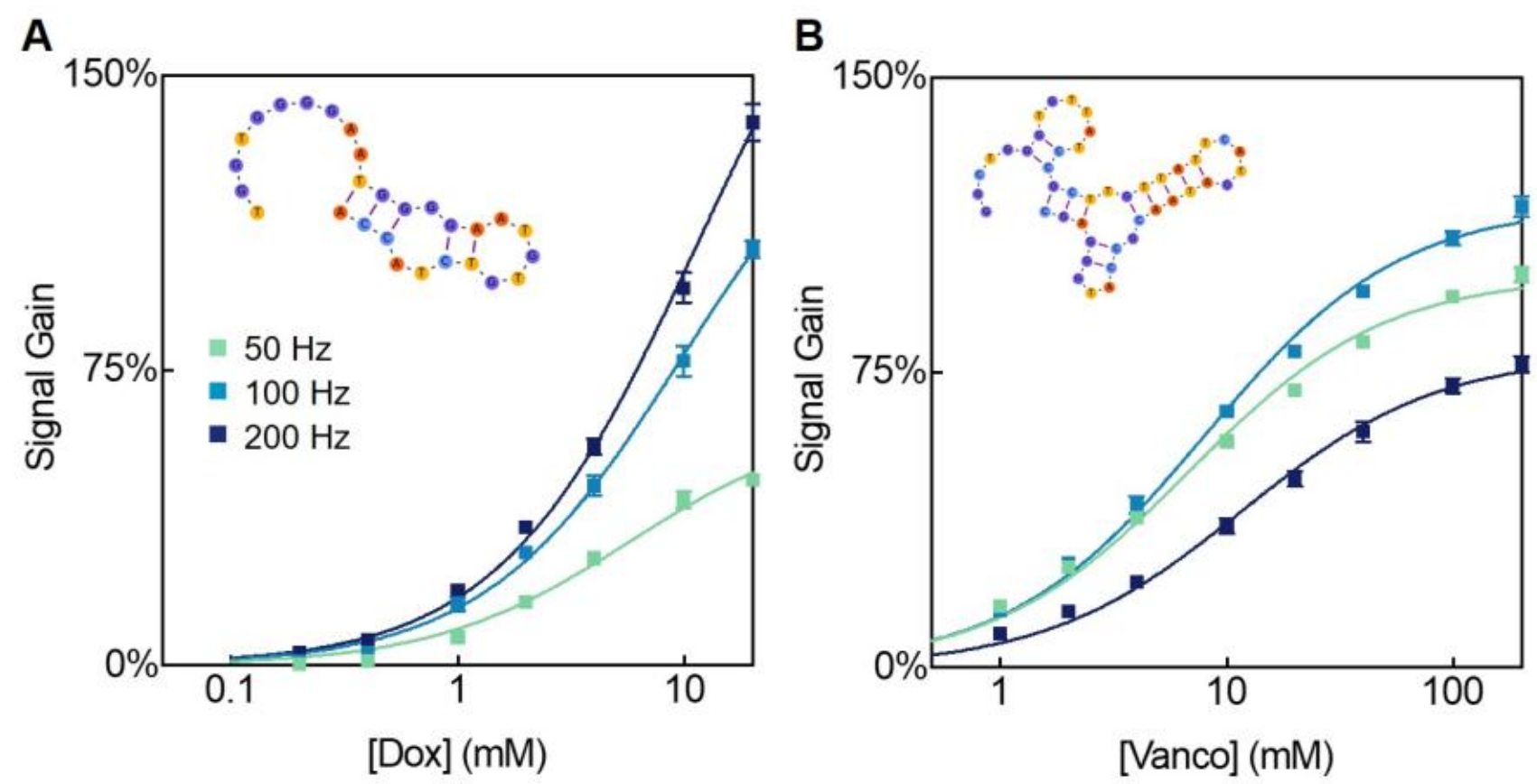

Fig. S7. Calibration curves of BICEP sensors against multiple analytes. We used our BICEP sensors to detect **A**) doxorubicin and **B**) vancomycin in PBS by swapping the immobilized aptamer switches. The aptamer switch sequences used are shown in **Table 1**.

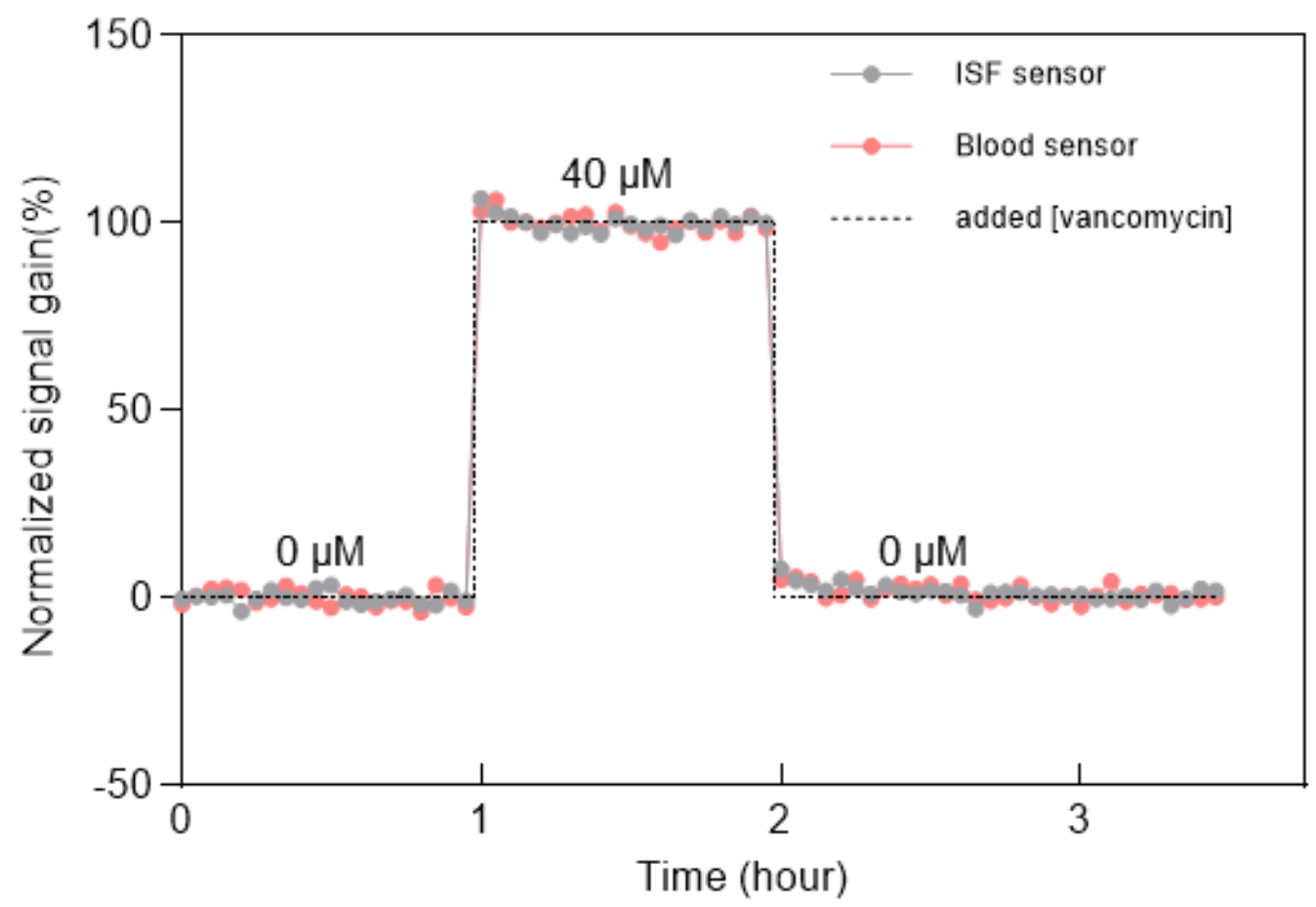

Fig. S8. Continuous monitoring with BICEP sensors demonstrates efficient mass transport and reversible binding kinetics. The dual-channel vancomycin-sensing BICEP sensor shank was sequentially exposed to three conditions: PBS buffer for one hour, followed by 40 µM vancomycin for one hour, and then re-exposed to PBS after two washes. Throughout this process, continuous square-wave voltammetry (SWV) was performed at a sampling interval of 3 min.

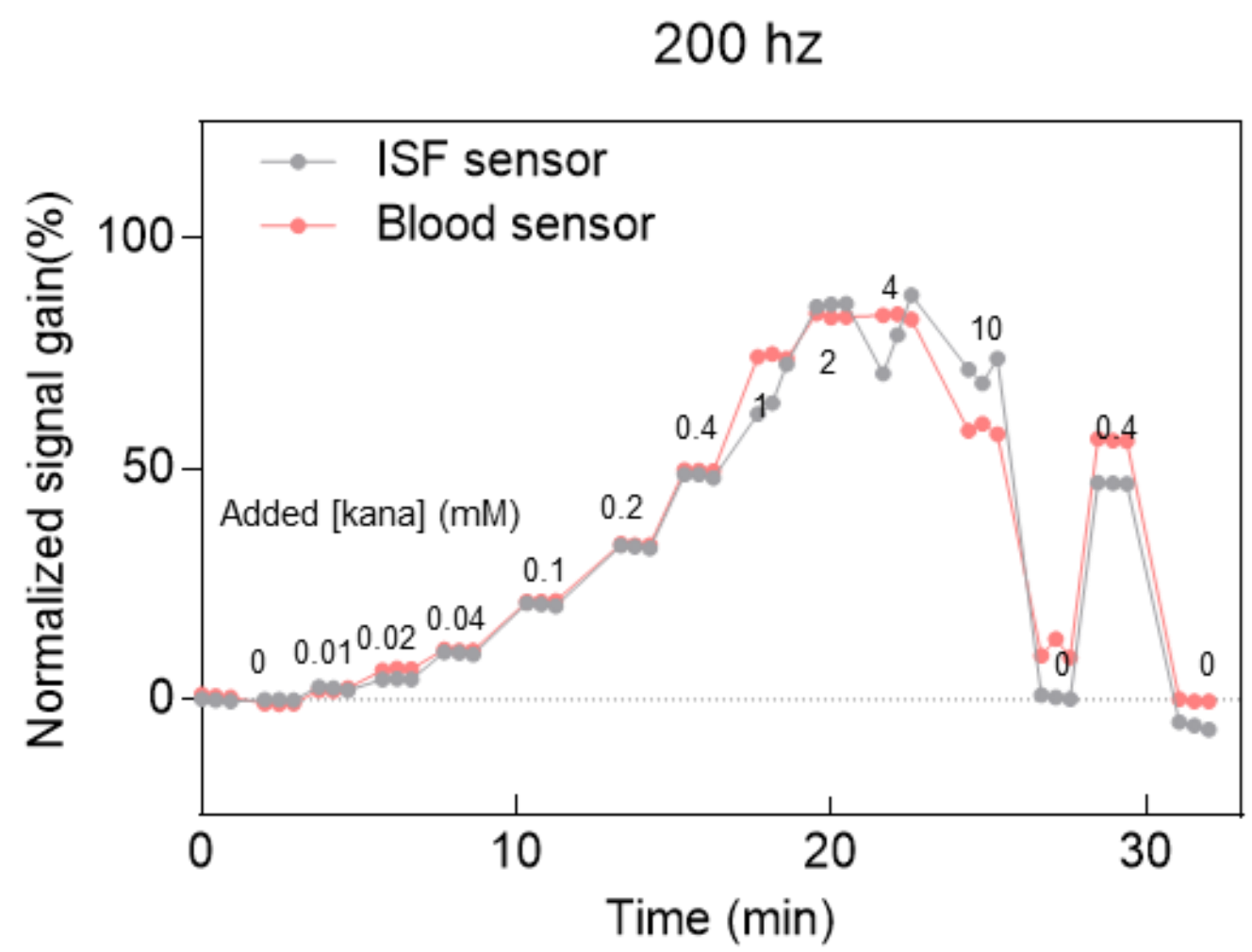

Fig. S9. Real-time dual-monitoring of kanamycin spike-in and washout in PBS using the integrated blood and ISF sensors on the same BICEP shank at 200 Hz SWV.

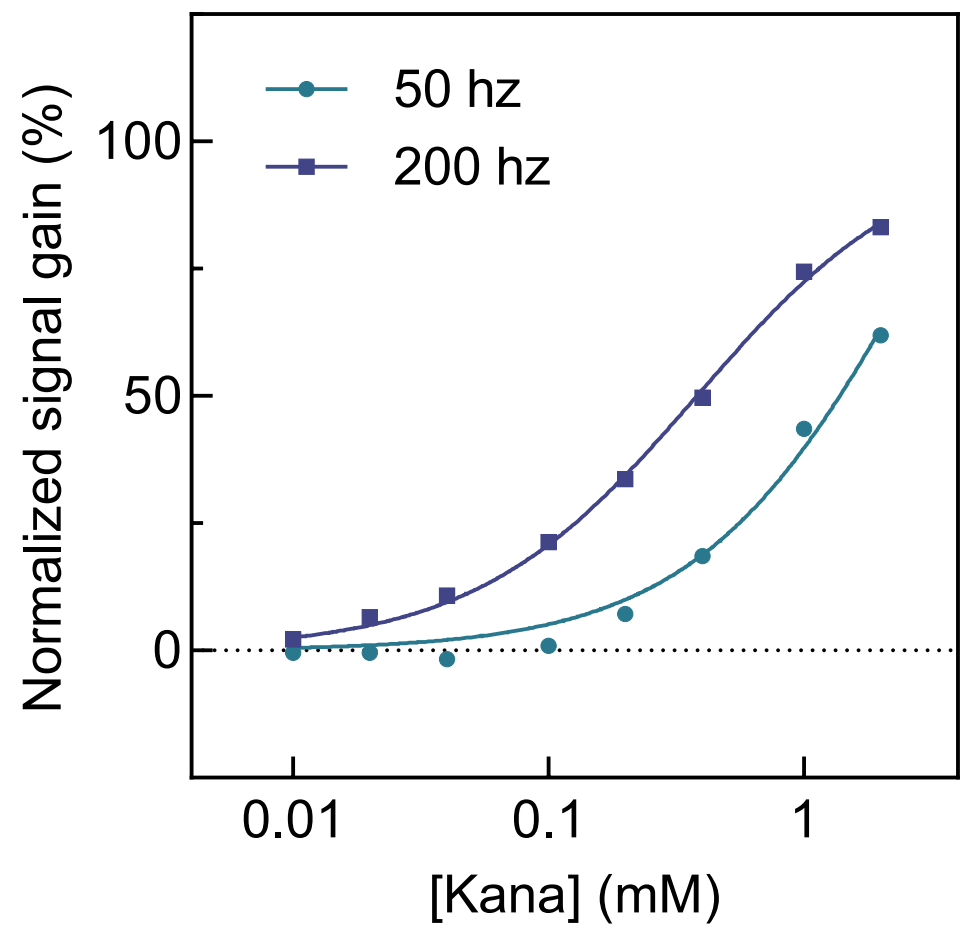

Fig. S10. SWV calibration curves at different frequencies from the same sensor. Signals were normalized to the maximum binding signal (B_{max}) based on Langmuir isotherm fitting. Data points and error bars respectively represent the mean and standard deviation of three consecutive measurements.

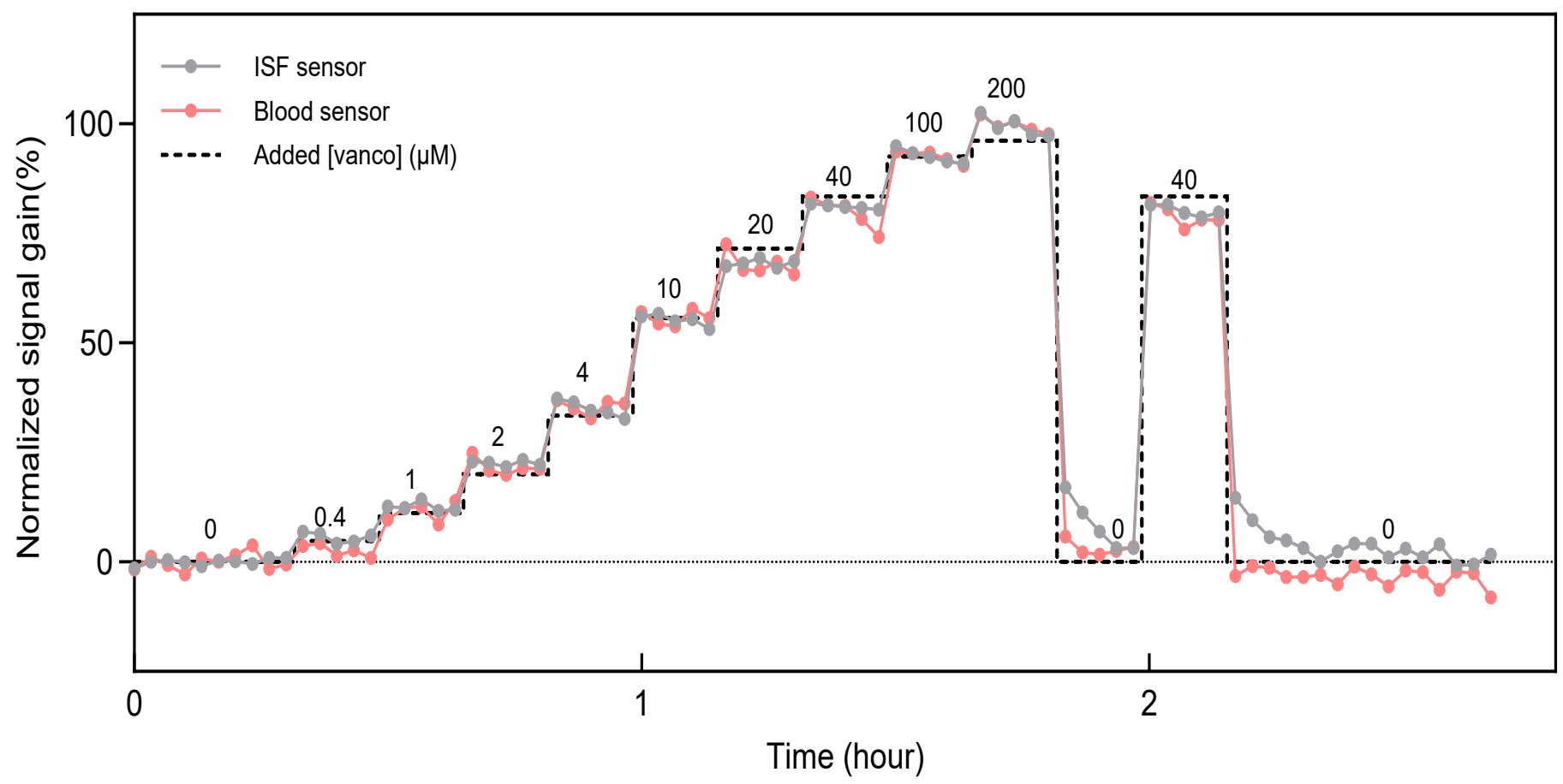

Fig. S11. Dual-compartment vancomycin monitoring with the BICEP shank in PBS. Real-time monitoring of vancomycin spike-in and washout in PBS using the integrated blood and ISF sensors on the same BICEP shank at 100 Hz SWV.

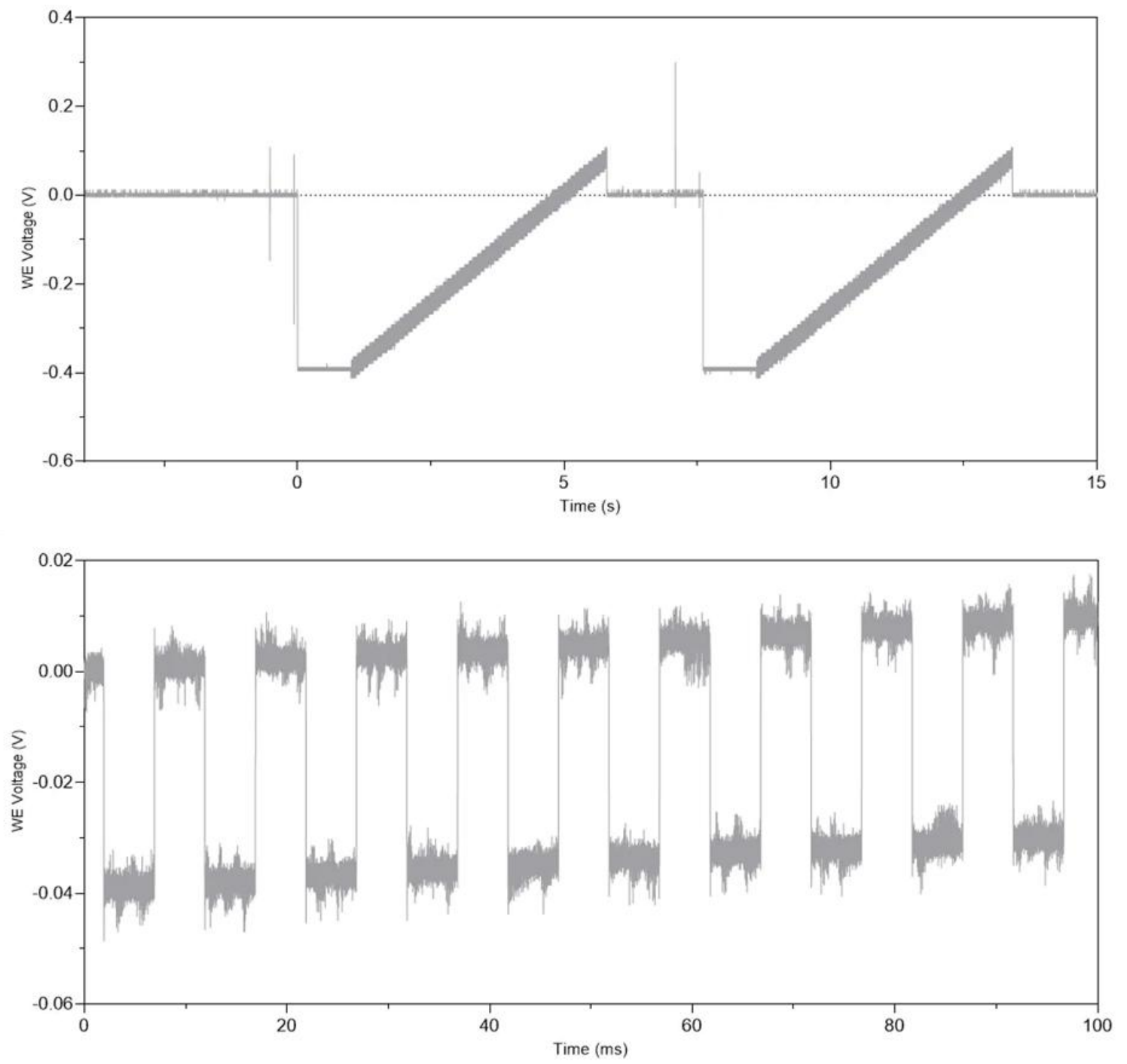

Fig. S12. Example of SWV potential waveform output from the BICEP wearable unit. The wireless potentiostat executed two consecutive SWV scans on dual channels, with a potential range of –400 mV to 80 mV, 1 mV step size, 20 mV amplitude, and 100 Hz frequency. The waveforms were recorded using an oscilloscope (Tektronix). The bottom panel provides a zoomed-in view of a 100-ms segment during the SWV scan, illustrating the detailed waveform structure.

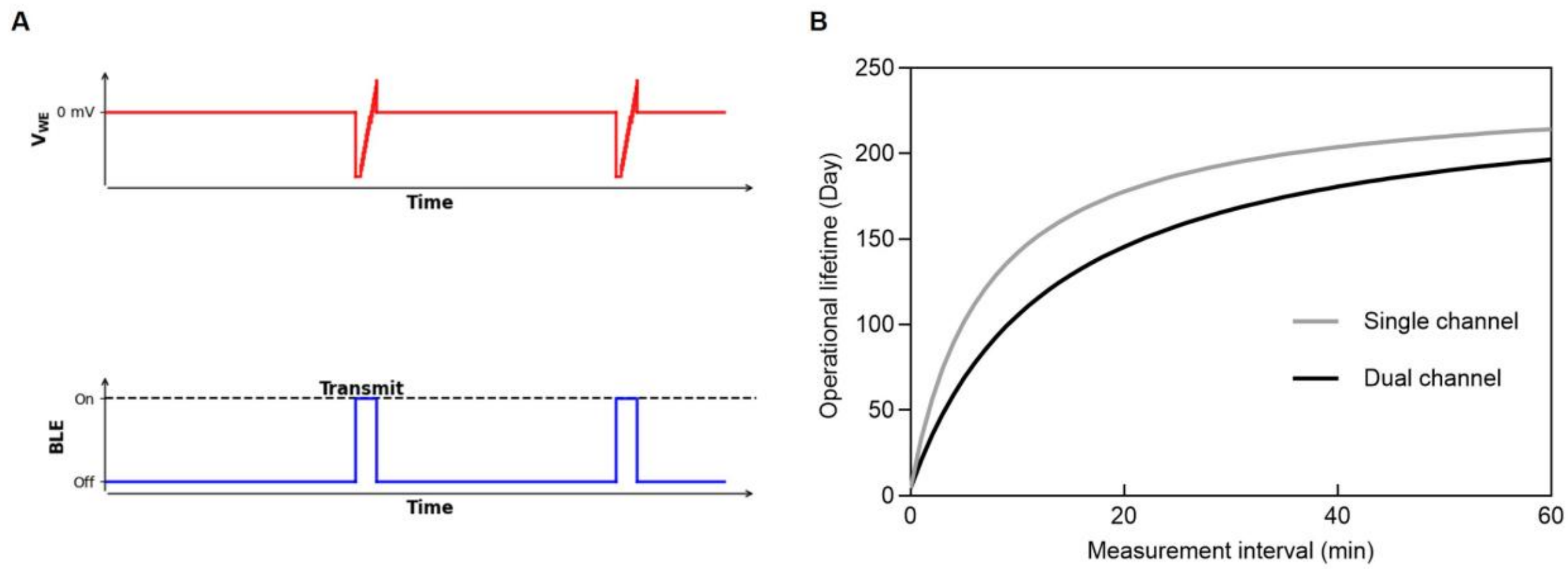

Fig. S13. Automatic "run-sleep" state switching of the BICEP wearable unit, enabling extended operational lifetime. A) Illustration of applied voltage on the working electrode as a function of time, triggered by Bluetooth for continuous measurement. **B)** Simulated operation lifetime as a function of the set measurement interval. The lifetime was calculated using the equation:

$$I_{avg} = \frac{t_{run} \cdot I_{run} + t_{sleep} \cdot I_{sleep}}{t_{run} + t_{sleep}} \, , \, t_{lifetime} = SD \cdot \frac{B_{cap}}{I_{avg}} \qquad (S7)$$

BICEP's SWV has a typical run time (t_{run}) of 8 seconds for a single channel and 15 seconds for a dual channel. The active current (I_{run}) is 5.67 mA, the sleep current (I_{sleep}) is 0.104 mA, the typical CR2450 battery capacity (B_{cap}) is 620 mAh, and the battery self-discharge rate (SD) is 99%.

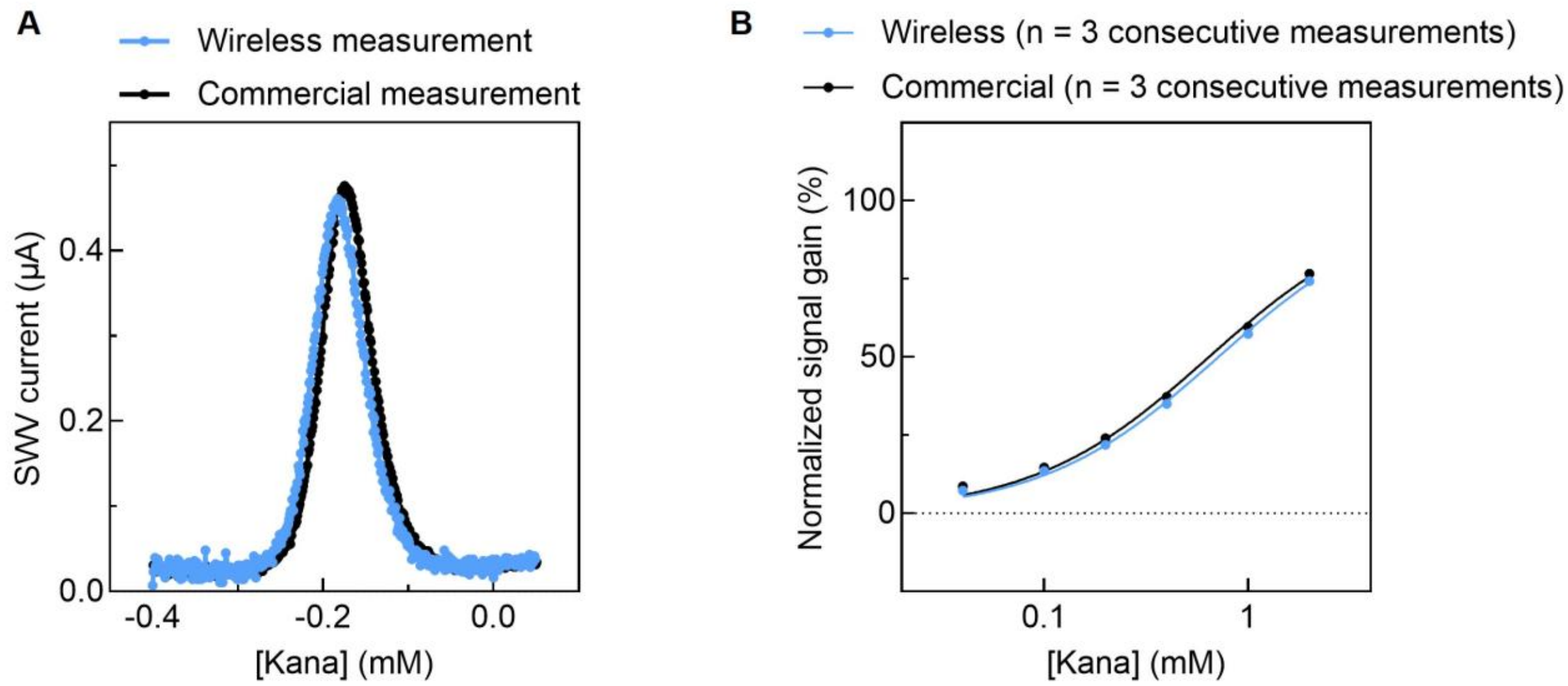

Fig. S14. Calibration of the wireless potentiostat against a commercial electrochemical workstation. (**A**) SWVs recorded with the same BICEP sensor in PBS using a commercial workstation (MultiEmStat4) and the custom wireless potentiostat under identical conditions (200 Hz SWV, −0.40 V to 0.05 V, 20 mV amplitude). (**B**) Kanamycin calibration curves obtained with each potentiostat by exposing the same BICEP sensor to 0.04–2 mM kanamycin in PBS. Data points and error bars represent the mean and standard deviation of three consecutive measurements.

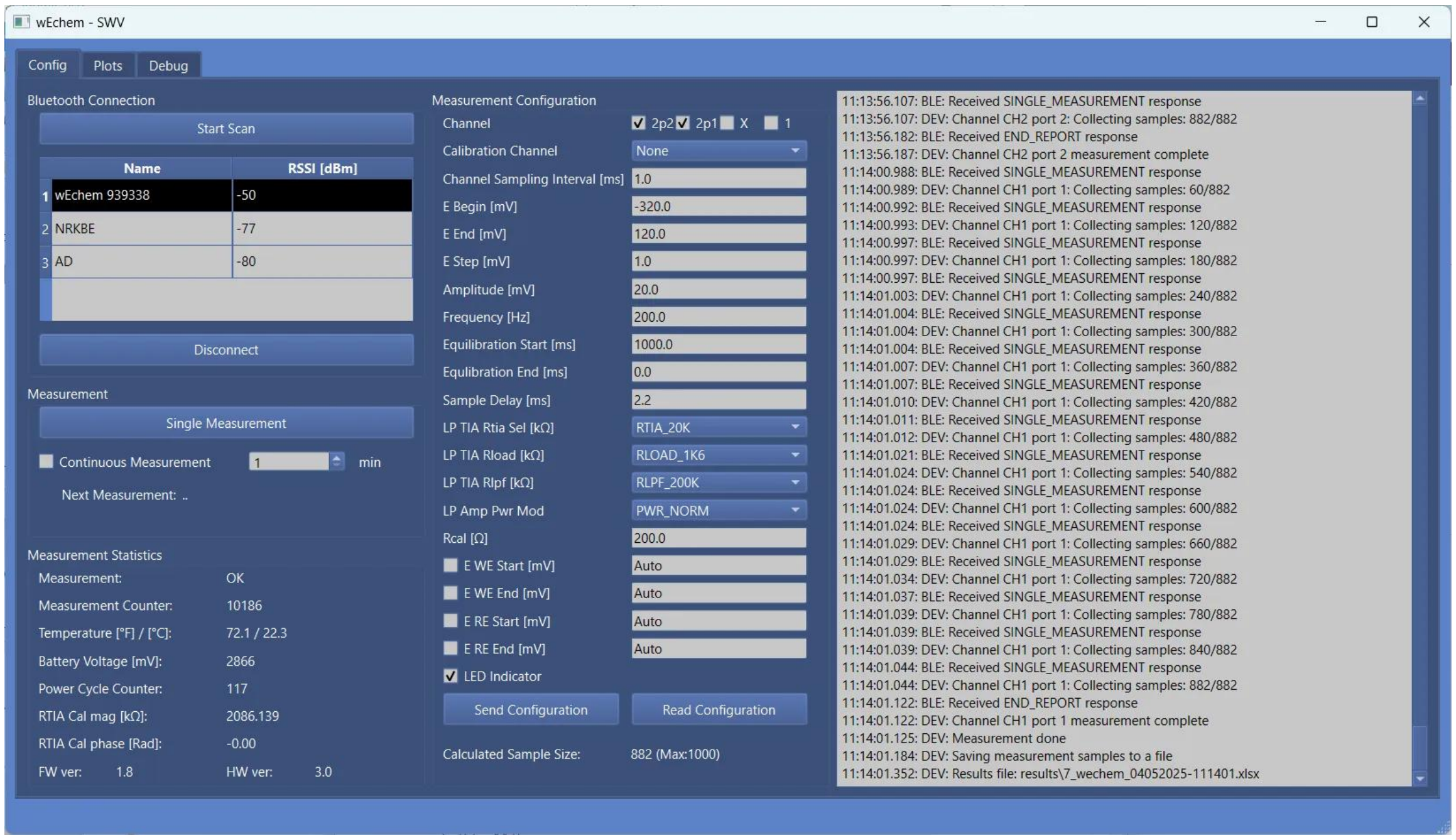

Fig. S15. Customized graphical user interface (GUI) for BICEP SWV configuration, measurement, and real-time monitoring. The left panel displays Bluetooth connection status, measurement frequency, and basic measurement statistics. The middle panel allows configuration of SWV parameters to be sent to the wireless potentiostat. The right panel shows Bluetooth communication logs. SWV waveforms and corresponding peak current time-lapse traces are visualized in the Plots tab.

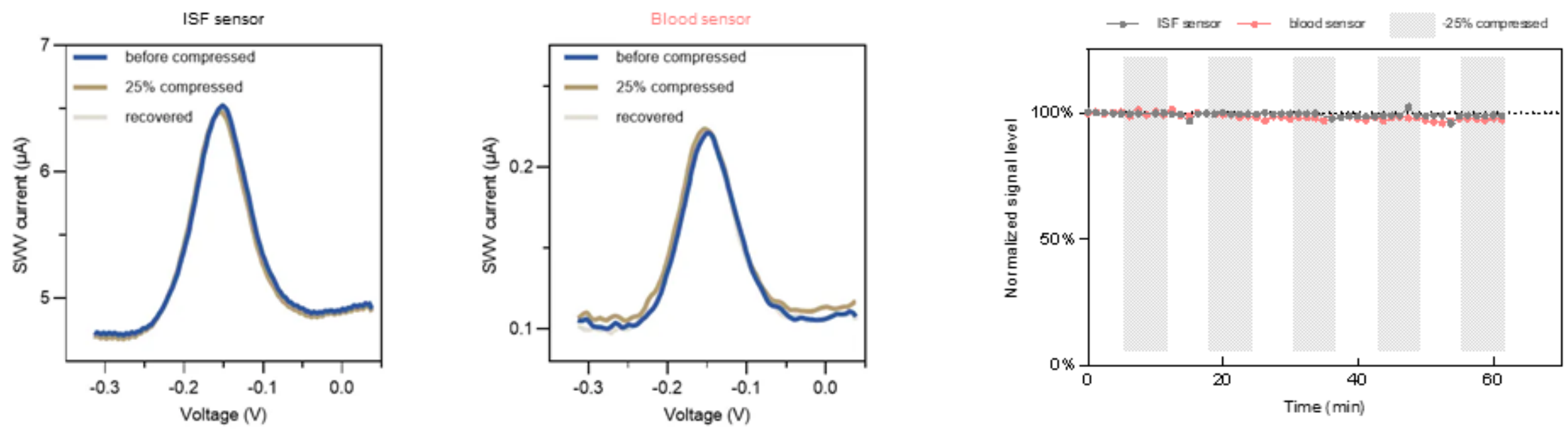

Fig. S16. Wireless monitoring of cyclic compressive strain effects on the BICEP readout. Raw SWV voltammogram data were acquired wirelessly while the dual-channel BICEP shank underwent cyclic compressive strain (-25%) in PBS. Both the ISF (left) and blood (middle) sensors showed minimal SWV signal degradation. The right panel summarizes the extracted SWV peak over five rounds of compressive strain with a minimal signal change of < 3%, demonstrating the mechanical stability of BICEP.

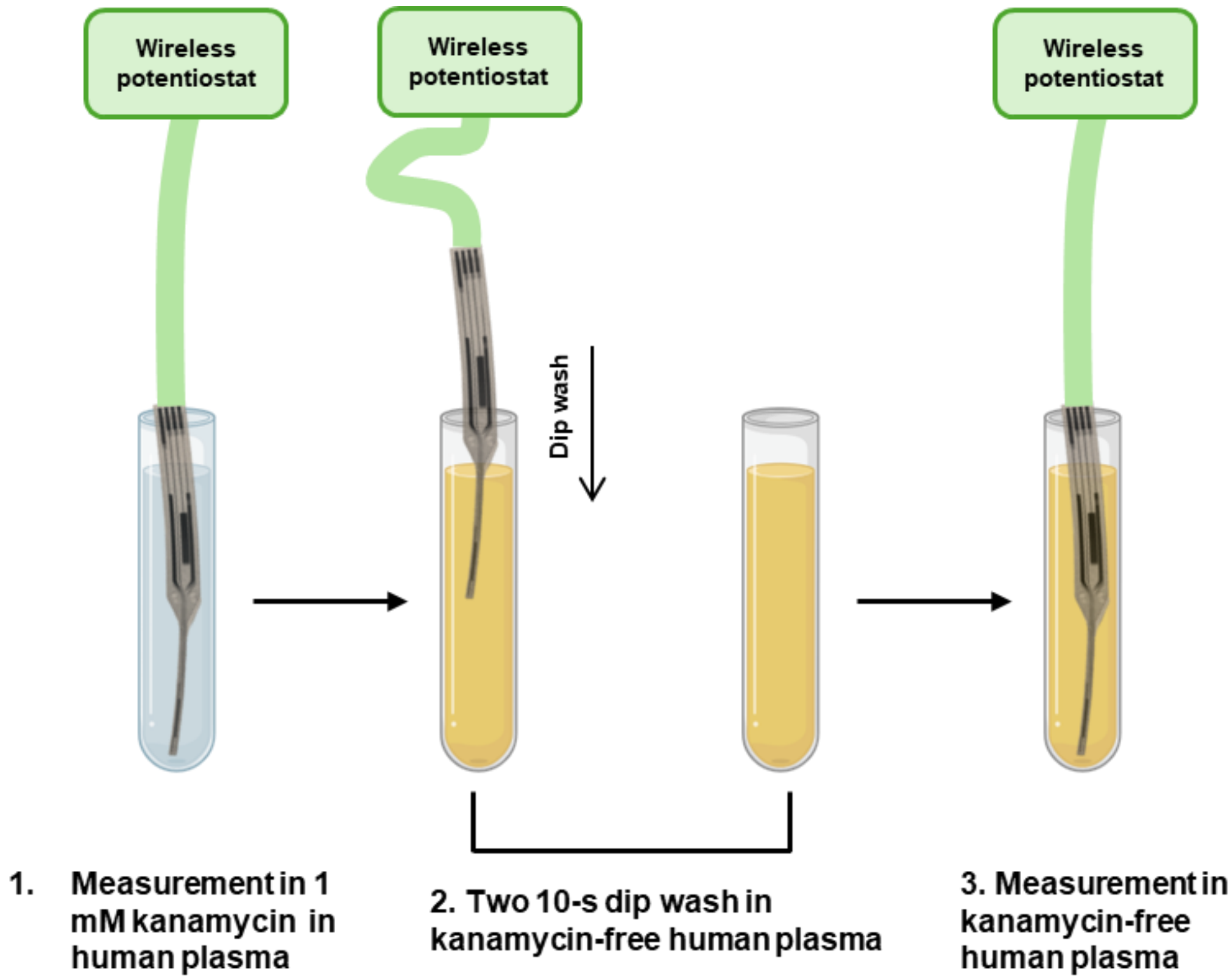

Fig. S17. Workflow for continuous BICEP monitoring in a static human plasma setup. We prepared 300 µL aliquots of pooled human plasma (kanamycin-free or spiked with 1 mM kanamycin) in PCR tubes and fully immersed the BICEP shank in each tube during wireless monitoring. At each concentration change step, we performed two brief dip-washes in kanamycin-free human plasma within ~20 seconds. We maintained an effective SWV acquisition frequency of one measurement every 1.2 min, with 0.2 min for SWV readout and about 1 min for manual dipping and tube exchange. Created in BioRender. hou, X. (2026) https://BioRender.com/l4ywcr1.

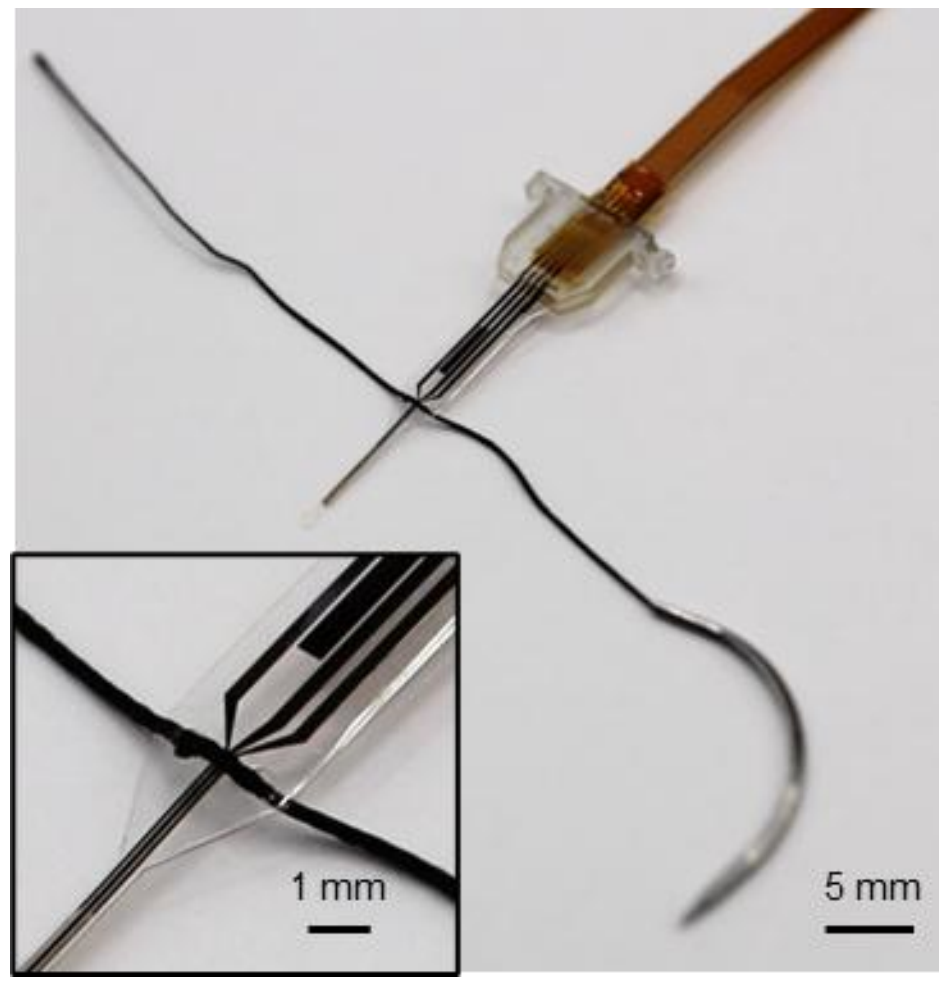

Fig. S18. The BICEP sensor shank secured with surgical sutures. The BICEP shank features two suture holes (0.4 mm diameter) located between the blood-sensing and ISF-sensing regions. A 4/0 surgical suture can be threaded through these holes to securely anchor the sensor shank to the tissue.

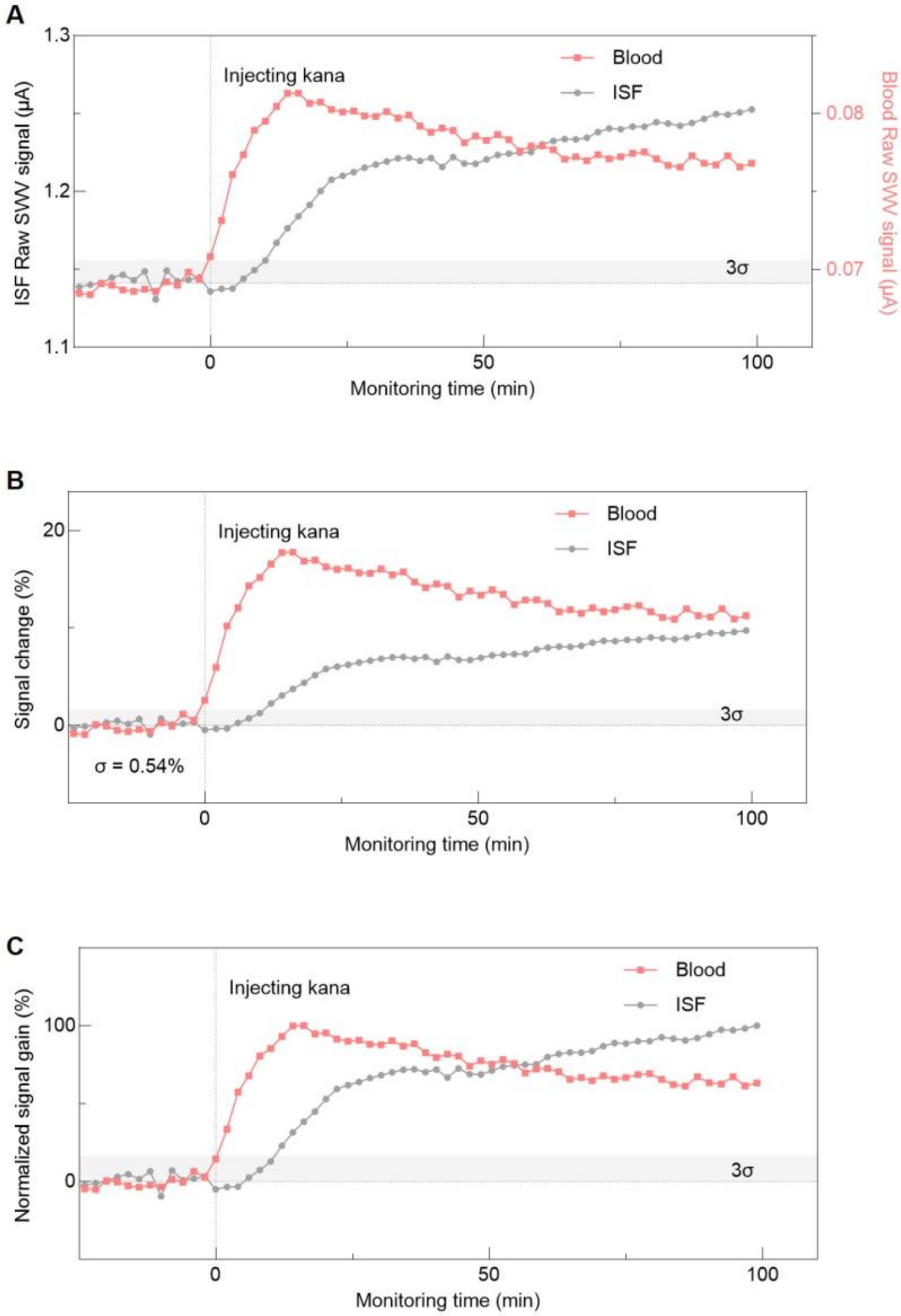

Fig. S19. Continuous blood–ISF monitoring with BICEP sensors and the real-time decision to administer kanamycin. (**A**) Continuous SWV peak extraction using the BICEP shank in a rat, as shown in **Figure 4B**. (**B**) We continuously extracted SWV peak amplitudes over time and then computed a running average during animal experiments. Once the baseline variation was < 2% over a 10-minute window relative to this running average (standard deviation = 0.54% in the example shown), a single 40 mg/kg bolus of kanamycin was administered via retro-orbital injection (dashed line). (**C**) Post-hoc visualization of continuous blood–ISF monitoring traces normalized to the post-injection peak for each animal, facilitating comparison of onset and rise times.

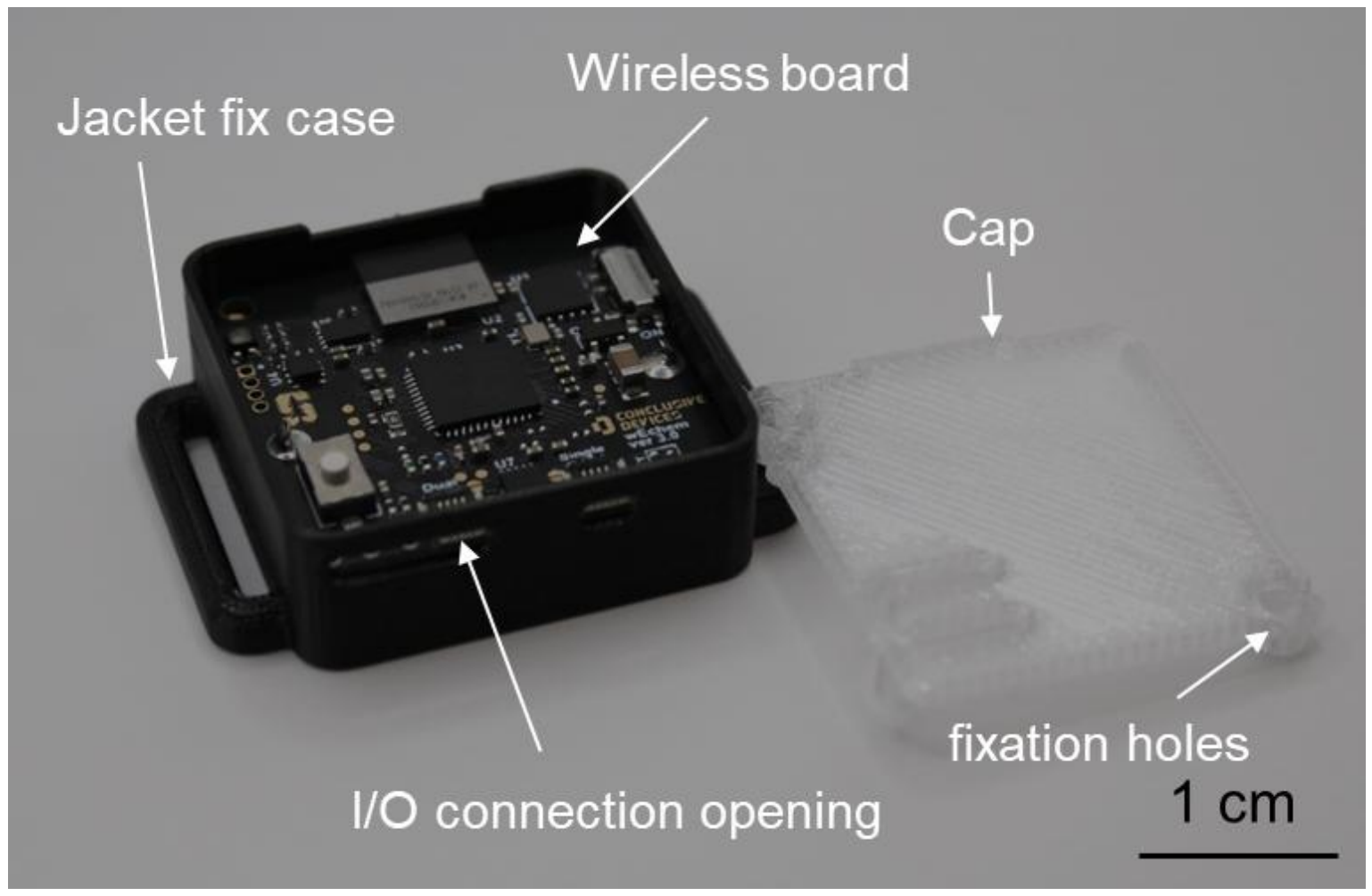

Fig. S20. Optical images of the BICEP wearable unit housed in a watch-sized 3D-printed case. The BICEP wireless board fits within the 3D-printed case, with the I/O port left open for either FPC or FFC connection. A clear cap can be mechanically fixed to the case to protect the board while still allowing the user to monitor its function (*e.g.*, via a blinking blue LED).

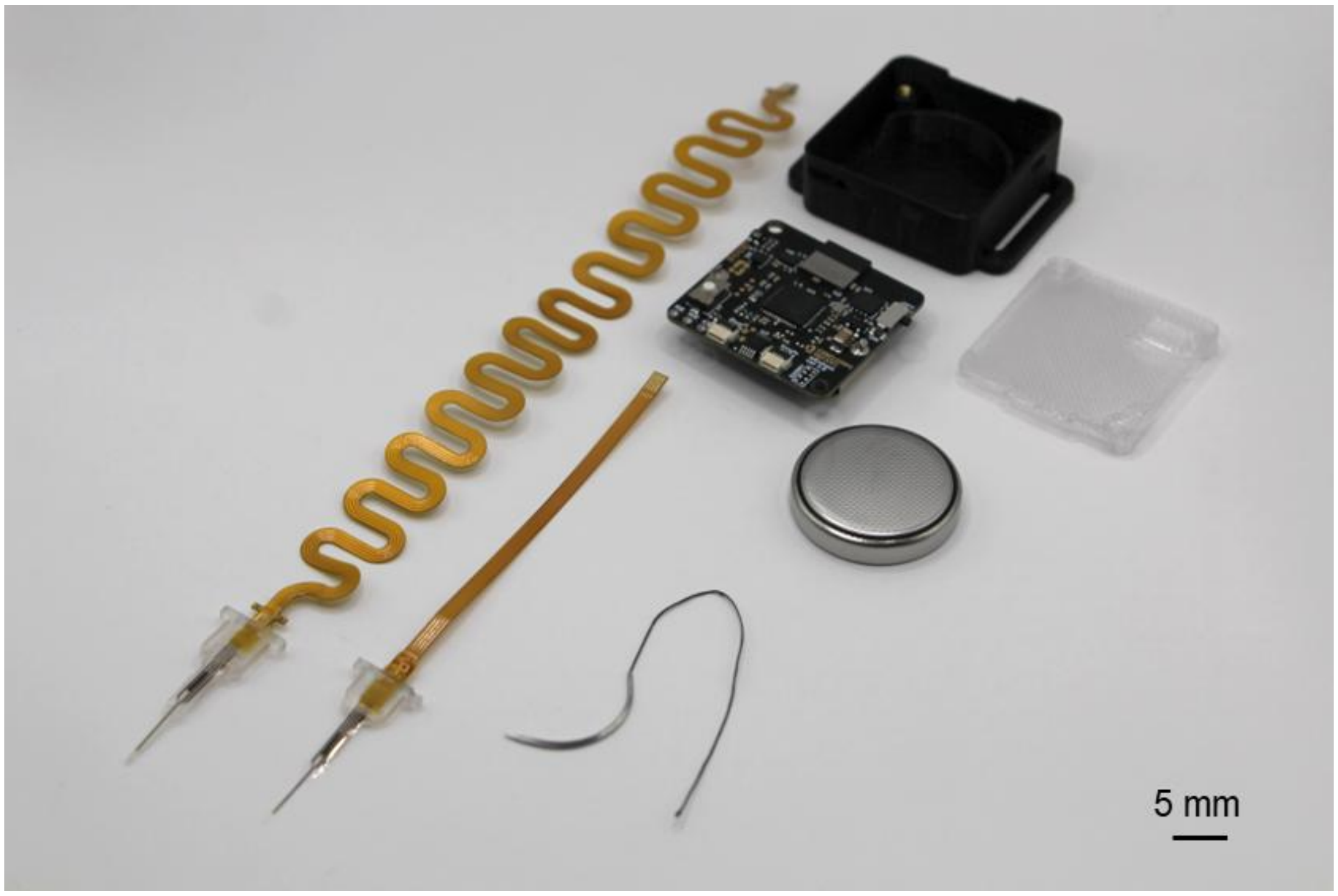

Fig. S21. The BICEP system for real-time, continuous monitoring of blood and ISF *in vivo*. The BICEP sensor shank is connected to a straight FPC for anesthetized studies, while a serpentine FPC enables flexible implantation for freely-moving studies. Both configurations use the same microfabricated sensor shank design. The wireless potentiostat connects directly to the FPC and is powered by a replaceable CR2450 coin cell battery, housed within a customized, watch-sized 3D-printed case. A 4/0 surgical suture is used to securely anchor the sensor shank in tissue.

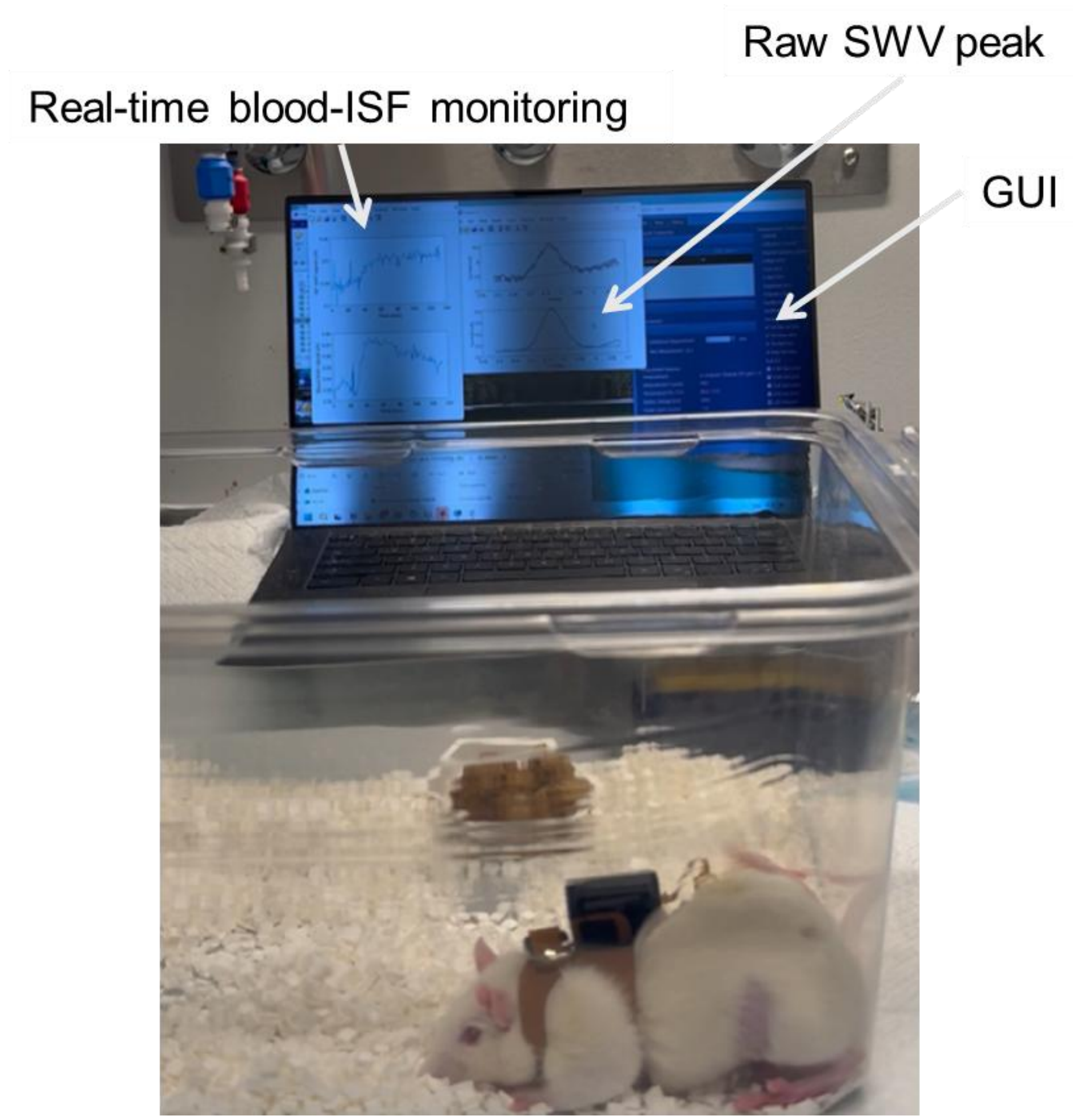

Fig. S22. BICEP system operation in a freely-moving rat. The BICEP sensor shank was surgically implanted in a rat, with the blood sensor positioned within the femoral vein and the ISF sensor placed on the fascia above the vein. The shank was connected to a wearable wireless potentiostat unit, enabling free movement of the rat. We used a custom GUI on a PC to remotely configure SWV parameters and continuously acquire signals from both the blood and ISF sensors. Custom MATLAB scripts extracted SWV peak currents and generated time-lapse traces of target signals in both compartments.

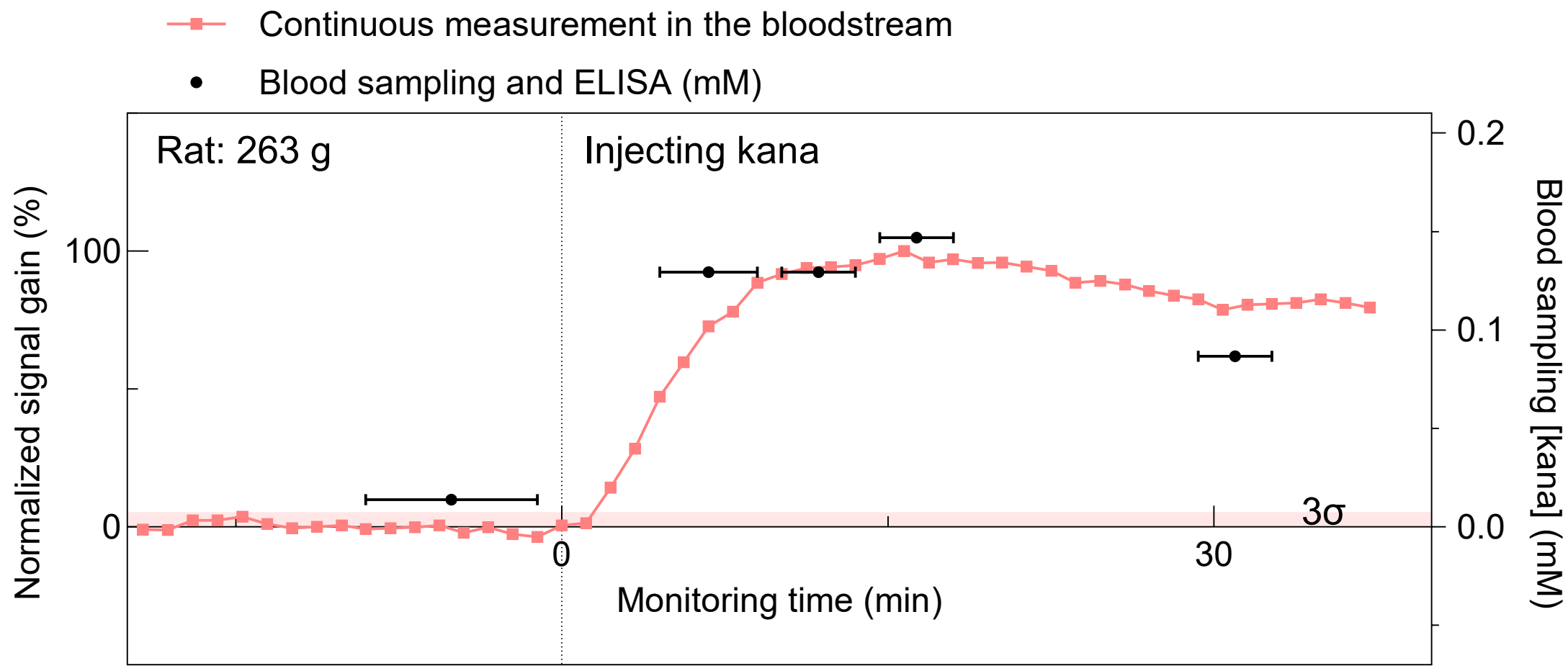

Fig. S23. Continuous blood monitoring with a BICEP sensor paired with episodic blood sampling and plasma ELISA quantification of kanamycin. We implanted a BICEP sensor in the rat femoral vein and continuously monitored blood kanamycin levels by SWV. We administered kanamycin (40 mg/kg) via retro-orbital injection at the time indicated by the dashed line. In parallel, we collected blood from the tail vein into Li-heparin tubes over the time windows indicated by the x-axis error bars to obtain sufficient volume for analysis and performed an ELISA to quantify plasma kanamycin.

Legends for movies

Movie S1. Blood-ISF monitoring in freely-moving rats with the BICEP system. A rat was implanted with the BICEP sensor shank, positioning the blood sensor in the femoral vein and the ISF sensor on the surrounding fascia. The animal carried a wearable wireless electrochemical board, enabling unrestricted movement throughout the monitoring period. We used a remote PC interface to configure SWV parameters and record continuous signals from both compartments. Real-time SWV data analysis was complemented by a MATLAB script that extracted peak currents and generated time-resolved signal traces from blood and interstitial fluid.

Movie S2. Conscious, freely-moving rats monitored by BICEP during unrestricted eating with both blood and ISF tracked.

Movie S3. Conscious, freely-moving rats monitored by BICEP during unrestricted grooming with both blood and ISF tracked.
